# Genome- and Transcriptome-wide Association Studies to Discover Candidate Genes for Diverse Root Phenotypes in Cultivated Rice

**DOI:** 10.1186/s12284-023-00672-x

**Published:** 2023-12-08

**Authors:** Shujun Wei, Ryokei Tanaka, Taiji Kawakatsu, Shota Teramoto, Nobuhiro Tanaka, Matthew Shenton, Yusaku Uga, Shiori Yabe

**Affiliations:** 1 Institute of Crop Sciences, National Agriculture & Food Research Organization, Tsukuba, Ibaraki 305-8518 Japan; 2 Institute of Agrobiological Sciences, National Agriculture & Food Research Organization, Tsukuba, Ibaraki 305-8604 Japan

**Keywords:** Root, Candidate gene search, Genetic variation, Association mapping, Genome-wide association study, Transcriptome-wide association study, Expression genome-wide association study

## Abstract

Root system architecture plays a crucial role in nutrient and water absorption during rice production. Genetic improvement of the rice root system requires elucidating its genetic control. Genome-wide association studies (GWASs) have identified genomic regions responsible for rice root phenotypes. However, candidate gene prioritization around the peak region often suffers from low statistical power and resolution. Transcriptomics enables other statistical mappings, such as transcriptome-wide association study (TWAS) and expression GWAS (eGWAS), which improve candidate gene identification by leveraging the natural variation of the expression profiles. To explore the genes responsible for root phenotypes, we conducted GWAS, TWAS, and eGWAS for 12 root phenotypes in 57 rice accessions using 427,751 single nucleotide polymorphisms (SNPs) and the expression profiles of 16,901 genes expressed in the roots. The GWAS identified three significant peaks, of which the most significant peak responsible for seven root phenotypes (crown root length, crown root surface area, number of crown root tips, lateral root length, lateral root surface area, lateral root volume, and number of lateral root tips) was detected at 6,199,732 bp on chromosome 8. In the most significant GWAS peak region, *OsENT1* was prioritized as the most plausible candidate gene because its expression profile was strongly negatively correlated with the seven root phenotypes. In addition to *OsENT1*, *OsEXPA31*, *OsSPL14*, *OsDEP1*, and *OsDEC1* were identified as candidate genes responsible for root phenotypes using TWAS. Furthermore, a *cis*-eGWAS peak SNP was detected for *OsDjA6*, which showed the eighth strongest association with lateral root volume in the TWAS. The *cis*-eGWAS peak SNP for *OsDjA6* was in strong linkage disequilibrium (LD) with a GWAS peak SNP on the same chromosome for lateral root volume and in perfect LD with another SNP variant in a putative *cis*-element at the 518 bp upstream of the gene. These candidate genes provide new insights into the molecular breeding of root system architecture.

**Supplementary Information:**

The online version contains supplementary material available at 10.1186/s12284-023-00672-x.

## Introduction

Rice (*Oryza sativa*) is an indispensable staple crop for sustainable food production in many Asian countries (Muthayya et al. 2014). Since roots play a crucial role in absorbing nutrients and water from the soil, root system architecture needs to be optimized to improve the yield, particularly in unfavorable environments (Gowda et al. 2011; Ahmadi et al. 2014). To genetically improve the root system architecture, the causal genes for root-related phenotypes should be identified and characterized. Although mutant analysis has identified several root-related genes in rice (Mai et al. 2014; Meng et al. 2019), statistical mapping is also a powerful approach for discovering beneficial alleles. Quantitative trait loci (QTL) mapping leverages natural variations to discover beneficial genes or alleles for molecular breeding, typically using a biparental population developed from two accessions with distinct traits. Some QTL for root phenotypes such as root growth angle, root thickness, and root length have been identified (Uga et al. 2011, 2012; Kitomi et al. 2015; Lou et al. 2015; Li et al. 2015), and two of them have been cloned (Uga et al. 2013; Kitomi et al. 2020). Although the genes and alleles identified in these studies have enhanced the molecular breeding of root phenotypes, the QTL mapping requires labor-intensive and time-consuming crossing, phenotyping, and genotyping, starting with biparental population development and ending with map-based cloning.

A genome-wide association study (GWAS) can identify genomic regions for the phenotype of interest using a diversity panel sequenced and genotyped for single nucleotide polymorphisms (SNPs) or insertion/deletion variants (indels), which is less laborious and time-consuming than creating a biparental population for QTL mapping. Beginning with pioneering studies using approximately 200 rice accessions with approximately 30,000 markers for root phenotypes measured in hydroponic cultivation systems (Clark et al. 2013; Courtois et al. 2013), GWAS have been conducted for various root phenotypes under different conditions and at different growth stages (Biscarini et al. 2016; Phung et al. 2016; Bettembourg et al. 2017; Wang et al. 2018a; Zhao et al. 2018, 2021a, b; To et al. 2019; Xu et al. 2020; Zhang et al. 2020; Anandan et al. 2022; Teramoto et al. 2022; Xiang et al. 2022; Hanlon et al. 2023). However, it is difficult to identify small-effect loci for root phenotypes based only on GWAS because the sample size is often limited as the evaluation of root phenotypes requires digging up the root from the soil, which has extremely low throughput.

The transcriptome, an intermediate phenotype that reflects complex genetic responses to the ambient environment, as well as genetic variation among accessions, presents a new opportunity to reveal the genetic control of terminal and/or fitness-related phenotypes (Kremling et al. 2018, 2019; Groen et al. 2020, 2022). Using the same statistical model as the GWAS, we can statistically test the association between the expression profile of each gene and the phenotypic value (transcriptome-wide association study, TWAS). TWAS has an advantage over GWAS in terms of statistical resolution for identifying candidate genes because linkage disequilibrium (LD) does not affect expression profile (Kremling et al. 2019; Li et al. 2021a). Owing to affordable RNA-seq technologies, TWAS has become a popular statistical mapping approach for various crop species such as maize (Hirsch et al. 2014; Lin et al. 2017; Kremling et al. 2019; Hershberger et al. 2022; Wu et al. 2022), sorghum (Ferguson et al. 2021; Pignon et al. 2021), soybean (Li et al. 2021a), rapeseed (Harper et al. 2012; Lu et al. 2014; Tang et al. 2021), and rice (Zhang et al. 2018; Liu et al. 2022). Although most studies have focused on the phenotype and expression profiles quantified in shoots or seeds, the TWAS has also been applied to rice root phenotypes. Lou et al. (2017) performed TWAS using 40,122 transcripts quantified in the roots of 37 rice accessions and identified several genes related to energy metabolism, production, and consumption that shape the deep or shallow root system architecture. In addition to TWAS, differential expression and gene ontology (GO) enrichment analyses have been applied to root transcriptome data to elucidate the molecular mechanisms of rice root system architecture (Takehisa et al. 2012; Kawakatsu et al. 2021). The results of these studies encourage using transcriptome data to accelerate candidate gene searches responsible for root phenotypes.

The expression profile of the candidate genes from the TWAS can further be statistically associated with genome-wide DNA polymorphisms, which helps us combine the GWAS and TWAS results. Considering the expression profile as the response variable of GWAS or QTL mapping, it is possible to identify a genomic region that regulates the expression profile of the gene of interest. This statistical mapping is called expression GWAS (eGWAS) or expression QTL (eQTL) mapping, and has been used to reveal the genomic basis of transcriptome variations in rice (Wang et al. 2010, 2014a; Horiuchi et al. 2015; Kuroha et al. 2017; Campbell et al. 2020; Kashima et al. 2021; Liu et al. 2022). Significant variants in eGWAS can be classified into *cis* or *trans*-effects according to the physical and/or genetic distance to the gene tested in the eGWAS (Wittkopp et al. 2004; Kliebenstein 2009). Interestingly, the *cis*-effects tend to be stronger than the *trans*-effects in several species, such as rice (Wang et al. 2010, 2014a; Liu et al. 2022), maize (Wang et al. 2018c), and lettuce (Zhang et al. 2017). Several studies have integrated eGWAS with GWAS and TWAS to explore candidate variants for the phenotype of interest by investigating the colocalization (overlap) between GWAS and *cis*-eGWAS peaks (Liu et al. 2022; Wu et al. 2022).

Taken together, comprehensive statistical mapping using both genome and transcriptome data is a promising approach for identifying candidate genes responsible for root phenotypes. As a compact set to efficiently investigate the high genetic diversity of rice, the World Rice Core Collection (WRC) was developed and recently resequenced (Kojima et al. 2005; Tanaka et al. 2020). Our previous study quantified the expression profiles and root phenotypes of 57 accessions of the WRC, showing subpopulation-specific stress response mechanisms (Kawakatsu et al. 2021). In this study, we applied GWAS and TWAS to the 12 root phenotypes in these 57 rice accessions to identify novel candidate genes responsible for the natural variation of root system architecture in rice using the available genome, transcriptome, and root phenotype datasets from previous studies (Tanaka et al. 2020; Kawakatsu et al. 2021). Furthermore, eGWAS was applied to the candidate genes from the TWAS, and the eGWAS and GWAS peaks were compared to identify a variant related to the expression profile of the candidate genes responsible for root phenotypes. We identified six candidate genes responsible for the root phenotypes using three statistical mappings.

## Materials and Methods

### Plant Materials and Field Trial for Phenotyping and Sampling

All statistical analyses were performed on the phenotypic values quantified by Kawakatsu et al. (2021) without any additional mathematical calculations (such as no transformation was applied for any phenotype). Therefore, we provide a brief overview of plant materials, field experiments, and phenotyping methods.

In total, three replicates of 57 accessions from the WRC (Kojima et al. 2005) were evaluated in an upland field at the Institute of Crop Science (National Agriculture and Food Research Organization, Ibaraki, Japan; 36.0289 °N, 140.0997 °E) from June 5 to August 1, 2018. The ratio of deep rooting (RDR) was quantified using plastic mesh baskets by calculating the ratio of the number of crown roots penetrating the lower part of the mesh (53°–90° to the horizontal) to the total number of crown roots penetrating the entire mesh (Uga et al. 2009). Using the WINRIZO Pro 2017a software (Regent Instruments, Quebec, Canada), root length (RL), root surface area (RSA), root volume (RV), root diameter (RD), and the number of root tips (NRT) for both crown roots (> 0.2 mm diameter roots, represented by ‘_C’ suffix in the abbreviation) and lateral roots (< 0.2 mm diameter roots, represented by ‘_L’ suffix in the abbreviation) were measured from the root samples collected from the soil using the backhoe-assisted monolith method (Teramoto et al., 2019). Additionally, root dry weight (RDW) of samples dried at 80 °C for three days was measured. Further details of the field experiments and measurement methods are described in Kawakatsu et al. (2021) and Teramoto et al. (2019).

### Transcriptome Data Processing

Total RNA was extracted from the crown roots of three plants per accession from the same field experiment using the HighGI method (Yoshino et al. 2020), and equal amounts of three RNA samples extracted from the same accession were pooled before RNA-seq library preparation. RNA-seq libraries were sequenced on a single lane of S4 flow cells with paired-end 150-bp and unique dual index reads using Illumina NovaSeq6000 at Macrogen, Japan. Reads were mapped to the IRGSP-1.0 genome assembly and MSU7 gene models using STAR aligner (Dobin et al. 2013). Uniquely mapped read counts were quantified using featureCounts version 1.6.4 (Liao et al. 2014). This pipeline yielded a read count matrix of 55,986 genes for 61 accessions. Further details of RNA extraction, sequencing, read mapping, and read quantification methods have been described previously (Kawakatsu et al. 2021).

From the read count matrix, fragments per kilobase of exon per million read (FPKM) values were calculated based on the trimmed mean of M value normalization in the {edgeR} package version 3.38.1 (Robinson et al. 2010). As reported previously (Kawakatsu et al. 2021), the log_2_(FPKM + 1) value was calculated and defined as the expression profile. We defined the gene as not expressed if the expression profile was lower than 1. After calculating the expression profiles, four non-WRC lines were excluded from the dataset. Finally, we excluded 39,085 genes not expressed in more than 50% WRC accessions. This generated an expression profile matrix of 16,901 genes for the 57 WRC accessions.

### Genotype Data Processing

The WRC accessions were sequenced as described previously (Tanaka et al. 2020). The paired-end reads were mapped against Os-Nipponbare-Reference-IRGSP-1.0 (Kawahara et al. 2013) pseudomolecules using the bwa mem (Li and Durbin 2009), and the duplicates were removed using Picard MarkDuplicates (http://broadinstitute.github.io/picard/). Using the GATK Best Practices for germline SNP/indel discovery (Van del Auwera et al. 2013), 2,805,329 SNPs and 357,639 indels were obtained from all 69 WRC accessions after variant calling and filtering as described by Tanaka et al. (2020). In this study, indels were removed from the association analyses for simplicity.

Considering the small sample size (*n* = 57) for the GWAS, the statistical power to identify an association between allelic and phenotypic variations was expected to be low, particularly for SNPs with low minor allele frequency (MAF). Therefore, we applied stringent MAF-based filtering to the 57 accessions to retain SNPs with MAF > 10% using VCFtools version 0.1.16 (Danecek et al. 2011), which removed 526,700 SNPs and retained 2,278,629 SNPs. We applied an LD-based SNP pruning method using PLINK version 1.9 (Purcell et al. 2007; Chang et al. 2015) to remove highly collinear SNPs (pairwise LD; *r*^2^ > 0.99) within 100 kb by setting the step size of the sliding window to 100 variants. This pipeline generated a SNP genotype dataset comprising 427,751 SNPs for 57 WRC accessions.

### GWAS

GWAS analyzes the strength of the statistical relationship between SNPs and phenotypic values to identify the phenotype-associated genomic regions. GWAS was performed for each root phenotype using 427,751 SNPs in the 57 WRC accessions based on the mixed linear model using the *GWAS* function in the {rrBLUP} package (Endelman 2011). The genomic relationship matrix was calculated on the same SNP set using VanRaden’s first formula (VanRaden 2008) using the *A.mat* function in the {rrBLUP} package. As the 57 accessions were divided into four or six subpopulations in Kawakatsu et al. (2021), we calculated the Bayesian information criterion (BIC) for the following three inclusion or exclusion models of the subpopulation as fixed covariates: (i) without subpopulation; (ii) with four subpopulations of admixed (*n* = 7), *aus* (*n* = 19), *indica* (*n* = 21), and *japonica* (*n* = 10); and (iii) with six subpopulations by dividing the 10 *japonica* accessions into *admixed-japonica* (*n* = 3), *temperate*-*japonica* (*n* = 3), and *tropical- japonica* (*n* = 4). The BIC value in the mixed model was computed based on the likelihood described by Kang et al. (2008), implemented in our in-house R script. The model with the lowest BIC value was selected as the optimal statistical model for each phenotype (Additional File 1: Table S1). Manhattan and quantile-quantile (QQ) plots were drawn by the {qqman} package (Turner 2018), and the false discovery rate-adjusted (FDR-adjusted) *P*-values were calculated by the *p.adjust* function using the “fdr” option.

To define the peak loci, we considered the physical distance and LD among the significant SNPs for each root phenotype. First, SNPs with FDR-adjusted *P*-value < 0.10 were defined to be significantly associated with the phenotype. Then, all significant SNP pairs within 100 kb showing pairwise LD (*r*^2^) > 0.50 were merged as a single peak locus, assuming that those SNPs were likely to be in LD with the same causal variant.

LD analyses were performed at both the regional and genome-wide scales to define a reasonable genomic region to search for a plausible candidate gene responsible for each GWAS peak. First, the *r*^2^ statistic was calculated for all SNP pairs within 500 kb of the peak SNP and visualized as a heatmap using the {LDheatmap} package (Shin et al. 2006). We also calculated genome-wide LD decay using the default method of PopLDdecay software (Zhang et al. 2019) on all 57 WRC accessions and each subpopulation. The LD-pruned 427,751 SNPs were used for the former regional LD analysis because showing too many SNPs in a heatmap is computationally difficult; meanwhile, the 526,700 SNPs before applying LD-pruning (after the MAF-based filtering on the 57 WRC accessions) were used for genome-wide LD decay analysis. According to the results of the LD diagnoses, the search interval for the candidate gene in the GWAS was set to ± 250 kb from the peak SNP (details are shown in the Results section). We used RAP-DB (Sakai et al. 2013) to obtain annotation information (version: IRGSP-1.0, 2022-09-01) for the genes in the search interval.

To further prioritize the candidate genes from the search interval, we tested the statistical dependence between the expression profile of the candidate genes in root samples and the genotype of the GWAS peak SNP using an analysis of variance (ANOVA). For each GWAS peak locus, the expression profile of each gene in the ± 250 kb searching interval was used as the response variable if the gene was expressed in the root. The number of alternative alleles of the GWAS peak SNP (coded as a numerical variable assuming an additive effect) and subpopulation (coded as a four-class factor variable: *japonica*, *indica*, *aus*, or admixed) were included in the model as explanatory variables without considering the interaction between the two variables.

### TWAS and GO Enrichment Analysis

TWAS is expected to complement the candidate gene search in GWAS by testing the statistical relationship between expression profiles and phenotypic values. We performed the TWAS using a method similar to that used in previous studies (Kremling et al. 2019; Hershberger et al. 2022; Wu et al. 2022). First, the probabilistic estimation of the expression residuals (PEER; Stegle et al. 2012) analysis was applied to the matrix of the expression profiles of the 16,901 genes for the 57 WRC accessions to reduce the hidden variation caused by experimental confounders. The number of factors in the PEER analysis was set to five based on the visual identification of the “elbow” in the diagnosis plot of the factor relevance (Additional File 2: Figure S1). The statistical model for each root phenotype in the TWAS was identical to the BIC-based optimal model used in the GWAS, by replacing the SNP genotype matrix with the matrix of the residual values from the PEER statistical model. The “P3D” option was set to TRUE in the *GWAS* function, as the *P*-values were inflated in the TWAS result if the “P3D” option was set to FALSE (Additional File 2: Figure S2). To identify candidate genes from the TWAS, we first applied the same significance threshold as in the GWAS (FDR-adjusted *P*-value < 0.10). Furthermore, we investigated the annotation and literature of all genes included in the top 10 strongest associations for each phenotype so as not to miss associations that did not pass our significance threshold but were stronger than the others. Additionally, the Pearson’s correlation coefficient between the expression profile and phenotypic value was calculated for the top 10 genes using all WRC accessions, as well as for each subpopulation.

Since the root system architecture is assumed to be a complex phenotype controlled by many genes, we applied GO enrichment analysis to discover the biological processes strongly related to the genetic variation of the root phenotypes in the WRC panel. For each phenotype, we first extracted the MSU IDs of genes with the top 1% positive and negative associations. As the top 1% associations for NRT, RL, RSA, and RV highly overlapped, particularly within the four phenotypes measured at the same part of the root, we took union of the top 1% genes responsible for these four crown and lateral root phenotypes (RS_C and RS_L, respectively; RS stands for root size) (details are shown in the Results section). Gene enrichment for the GO term related to a biological process was tested for the top 1% gene sets using the *enricher* function in the {clusterProfiler} package with its default parameters (Yu et al. 2012; Wu et al. 2021), with the 16,901 genes expressed in roots as the reference set. The GO for each transcript was obtained from RAP-DB (“IRGSP-1.0_representative_annotation_2022-09-01.tsv”). To use the ontology data assigned to each transcript in RAP-DB, MSU-ID was converted to RAP-ID based on the ID converter file in RAP-DB (“RAP-MSU_2022-09-01.txt”). If MSU-ID and RAP-ID did not have a one-to-one correspondence, the gene was removed from the enrichment analysis. For each converted RAP-ID, the GO terms were obtained from all potential transcript IDs.

### eGWAS for the Candidate Genes in TWAS

To connect the results from TWAS and GWAS, we performed eGWAS for genes possessing the top 10 strongest associations with at least one root phenotype in the TWAS. The statistical method for the eGWAS, including BIC-based model selection, was identical to that used for the GWAS; however, the expression profile was considered as the response variable of the mixed model. If there was at least one significant (FDR-adjusted *P*-value < 0.10) SNP within 250 kb of the gene position in the eGWAS, the most significant SNP was defined as the *cis*-eGWAS peak SNP for the gene. As we did not detect any significant *trans*-eGWAS SNPs for any tested gene, we explained the method used to compare the GWAS and eGWAS results when a *cis*-eGWAS peak SNP was detected. We calculated the physical distance (bp) and pairwise LD (*r*^2^) between the *cis*-eGWAS peak SNP and the SNP with the lowest *P*-value on the same chromosome in the GWAS for each relevant phenotype with which the gene possessed the top 10 associations in the TWAS. If the two SNPs were closer than 250 kb and their pairwise LD was > 0.50, we considered the gene to have an overlapping peak between the eGWAS and GWAS. An overview of the analysis pipeline is provided in Additional File 2 (Figure S3).

For the genes eventually selected as overlapping candidates among the GWAS, TWAS, and eGWAS, SNPs and indels from 2 kb upstream to the end of the gene region defined by the MSU7 gene model were extracted from the polymorphic genotype dataset of 2,805,329 SNPs and 357,639 indels (Tanaka et al. 2020) to identify a potential *cis*-variant for the candidate genes. The SnpEff annotations of the extracted variants were investigated in the TASKE + database (Cingolani et al. 2012; Kumagai et al. 2019). The PLACE database (Higo et al. 1999) visualized in the JBrowse of RAP-DB (Sakai et al. 2013) was also explored to determine whether any of the upstream variants disrupt a promoter motif.

## Results

### GWAS Identified Three Genomic Regions Responsible for Root Phenotypes

**Fig. 1:**
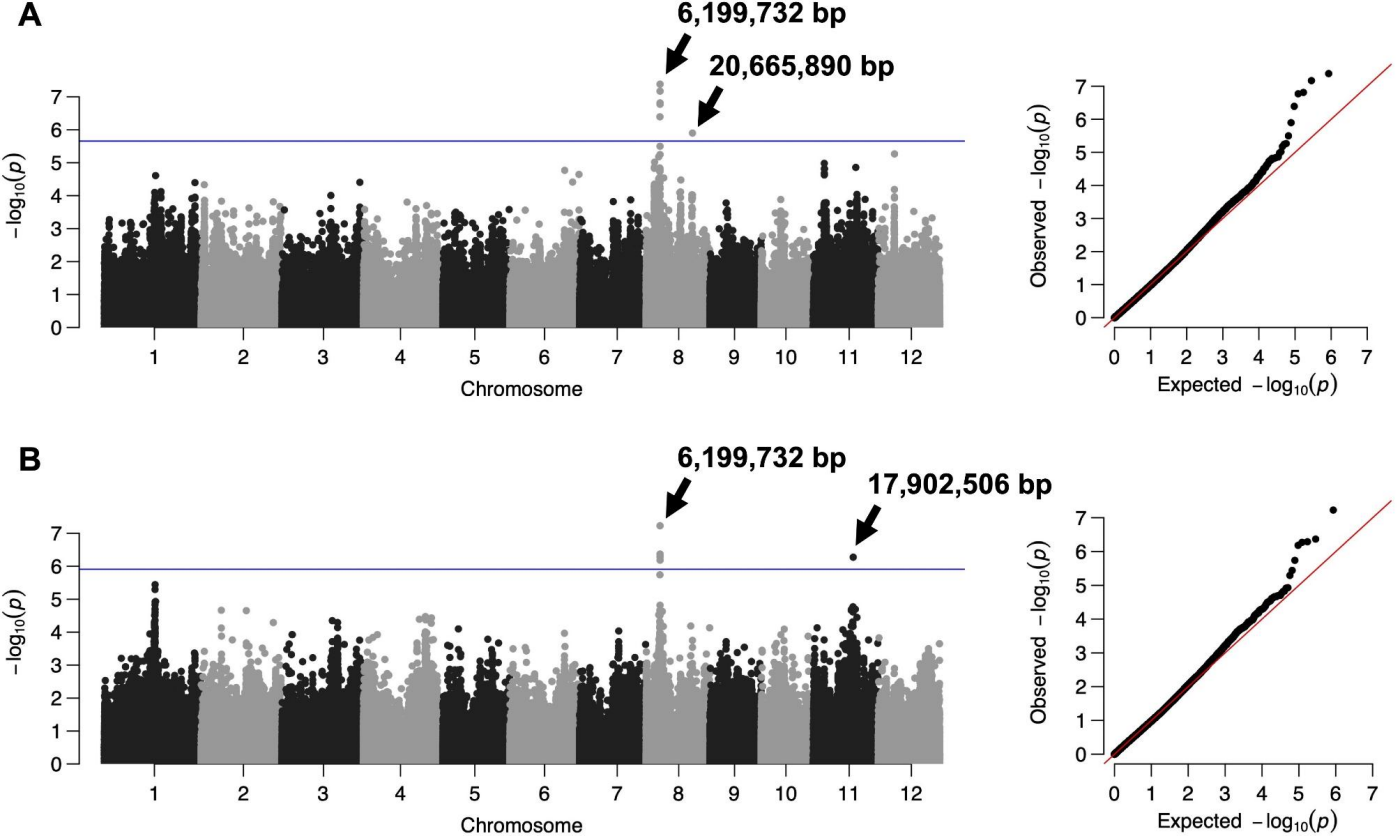
Manhattan and quantile-quantile plot for (**A**) RSA_C and (**B**) RSA_L. In total of three peak SNPs were identified for seven root phenotypes, including the two phenotypes shown as representative results in this figure. The blue horizontal line in the Manhattan plot represents the 10% FDR cutoff

To identify the candidate genes responsible for diverse root phenotypes, we conducted a GWAS for the 12 root phenotypes in 57 WRC accessions using 427,751 SNPs. Three peak SNPs with 10% FDR responsible for 7 out of the 12 phenotypes, causing 10 significant associations between SNPs and phenotypes, were detected (Table 1; Fig. 1; Additional File 2: Figure S4). The most significant peak SNP for seven root phenotypes (RL_C, RSA_C, NRT_C, RL_L, RSA_L, RV_L, and NRT_L) was identified at 6,199,732 bp on chromosome 8, with the highest − log*P* value (− log*P* = 8.07) for RL_C. The second peak SNP found at 20,665,890 bp on the same chromosome was significantly associated only with RSA_C (− log*P* = 5.90; FDR-adjusted *P*-value = 0.09). The third peak SNP at 17,902,506 bp on chromosome 11 was significantly associated with RSA_L (− log*P* = 6.27; FDR-adjusted *P*-value = 0.06) and RV_L (− log*P* = 6.50; FDR-adjusted *P*-value = 0.06; Fig. 1B). No significant associations were identified with the remaining five root phenotypes (RV_C, RDR, RDW, RD_C, and RD_L; Additional File 2: Figure S4).

**Table 1:** Summary of the 10 significant associations detected in GWAS

Chromosome	Peak SNP position (bp)	Phenotype	Phenotype abbreviation	−log*P*	FDR-adjusted *P*-value
8	6,199,732	Crown root length	RL_C	8.07	0.004
8	6,199,732	Crown root surface area	RSA_C	7.38	0.014
8	6,199,732	The number of crown root tips	NRT_C	7.82	0.006
8	6,199,732	Lateral root length	RL_L	7.05	0.038
8	6,199,732	Lateral root volume	RV_L	7.32	0.020
8	6,199,732	The number of lateral root tips	NRT_L	6.85	0.061
8	6,199,732	Lateral root surface area	RSA_L	7.23	0.025
8	20,665,890	Crown root surface area	RSA_C	5.90	0.090
11	17,902,506	Lateral root surface area	RSA_L	6.27	0.056
11	17,902,506	Lateral root volume	RV_L	6.50	0.055

GWAS; genome-wide association study, FDR; false discovery rate

We analyzed both regional and genome-wide LD patterns to determine reasonable genomic intervals for screening the candidate gene(s) responsible for GWAS peaks. The pairwise LD (*r*^2^) within 500 kb around the peak SNP did not show any obvious LD blocks (Additional File 2: Figure S5), likely because of the high genetic diversity and small sample size of the WRC panel. When the *r*^2^ values between the peak SNP and others were visualized on a regional Manhattan plot, most of the SNPs in high and moderately high LD (*r*^2^ > 0.80 and > 0.60, respectively) with the peak SNPs were located within 60 and 250 kb of the peak SNP, respectively (Additional File 2: Figure S6). Additionally, the genome-wide LD decay was calculated, and the mean *r*^2^ value decreased to 0.23 in the entire WRC panel when the distance between SNPs was approximately 250 kb (Additional File 2: Figure S7). Based on the results of LD analyses, the search region for candidate genes was set to ± 250 kb from each peak SNP. A total of 70 (for the peak at 6,199,732 bp on chromosome 8), 61 (for the peak at 20,665,890 bp on chromosome 8), and 68 (for the peak at 17,902,506 on chromosome 11) MSU loci were detected within 250 kb of the peak SNP (Additional File 1: Table S2–S4).

**Fig. 2:**
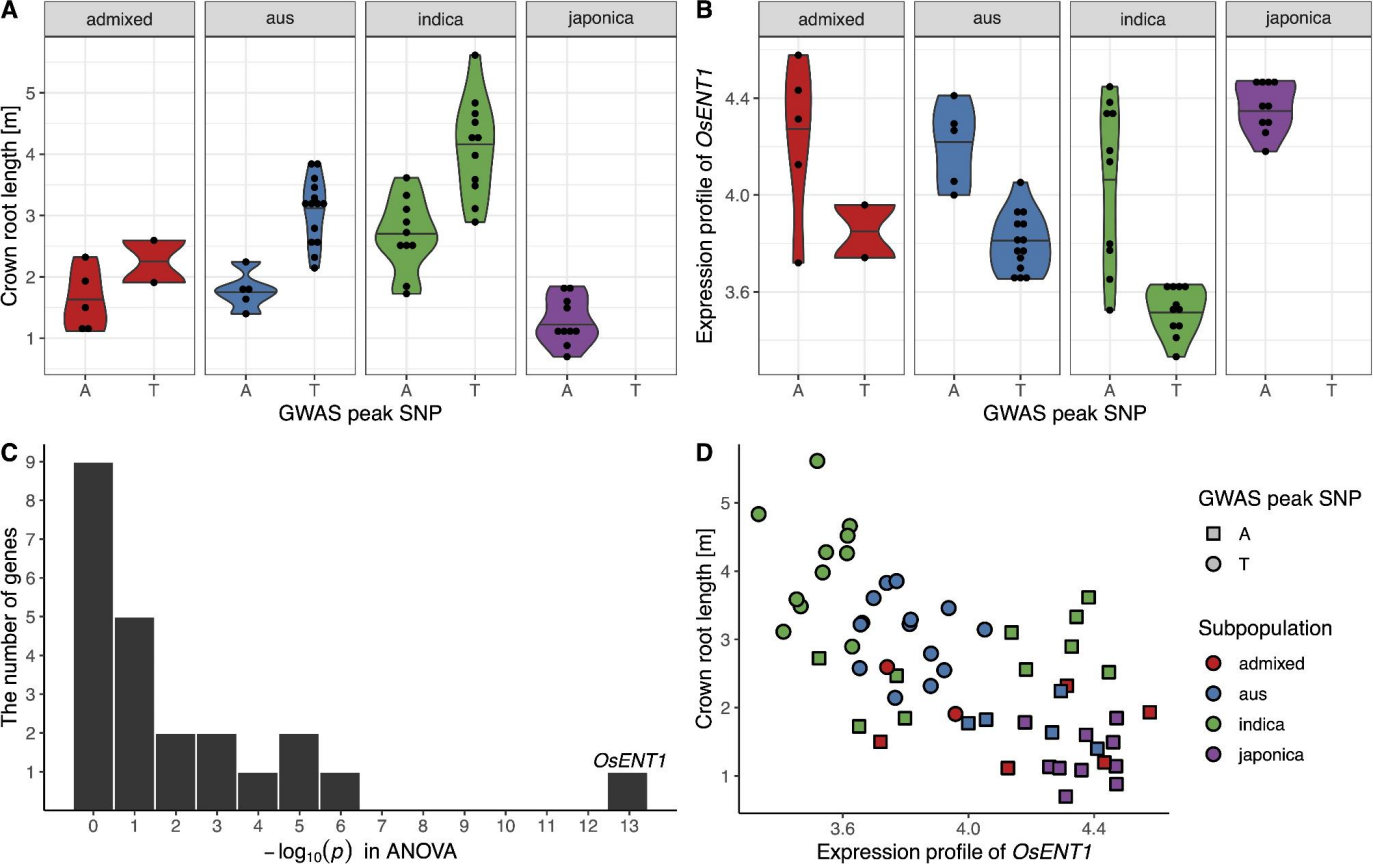
Relationship between *OsENT1* and the GWAS peak SNP at 6,119,732 bp on chromosome 8. We searched for the most plausible candidate gene for the GWAS peak SNP at 6,119,732 based on the root transcriptome data. The WRC accessions with the alternative allele (thymine) at the GWAS peak SNP had (**A**) longer crown root length and (**B**) lower *OsENT1* gene expression profile than those with the reference allele (adenine), respectively. (**C**) Histogram of the − log*P* values of the candidate genes around the peak SNP based on the ANOVA. (**D**) Scatter plot between *OsENT1* gene expression profile and crown root length. The horizontal bars in the violin plots represent median value

We further investigated candidate genes in the search region by leveraging their expression profiles in the root. An ANOVA between allelic variation and the expression profile of candidate genes expressed in the roots was conducted (Additional File 1: Table S2–S4), which revealed that the allelic variation of the most significant peak SNP was the most strongly related (*P* = 1.43 × 10^− 13^) to the expression profile of *OsENT1* gene (*LOC_Os08g10450*), which is located from 6,142,125 to 6,144,418 bp (approximately 55 kb from the GWAS peak SNP) and encodes an equilibrative nucleoside transporter (Fig. 2C). WRC accessions with the alternative allele (thymine) at this peak SNP showed longer RL_C and lower *OsENT1* expression profile than those with the Nipponbare reference type (adenine) in all subpopulations (Fig. 2A and B). Moreover, *OsENT1* expression profiles and RL_C phenotypic values were strongly negatively correlated (*r* = − 0.67; Fig. 2D). These results suggest that *OsENT1* is the most plausible candidate gene among the 70 candidates in the ± 250 kb region of the most significant GWAS peak responsible for seven root phenotypes including RL_C.

Except for the most significant peak SNP, the ANOVA did not find any other candidate genes with strong significance and an interpretable annotation. The top ANOVA hit gene for the second peak SNP was *LOC_Os08g33440* (*P* = 2.35 × 10^− 6^), which putatively encodes a protein similar to dihydrolipoamide S-acetyltransferase. Besides, *OsMADS23* (*LOC_Os08g33488*, *P* = 3.00 × 10^− 3^), which encodes a stress-responsive MADS-box transcription factor and functions as a positive regulator in response to osmotic stress by regulating ABA biosynthesis (Li et al. 2021b), was one of the 12 significant (*P* < 0.05) genes for the second peak SNP. The top ANOVA hit gene for the third peak SNP was *LOC_Os11g31110* (*P* = 2.42 × 10^− 3^), which was annotated as a conserved hypothetical protein.

### TWAS Suggested Five Novel Associations Responsible for Root Phenotypes

TWAS can extend GWAS-based candidate gene search by testing the statistical association between phenotypic values and expression profiles instead of the SNP genotype. We applied TWAS for the 12 root phenotypes on the 16,901 genes expressed in the root and identified six significant statistical associations under the threshold of FDR-adjusted *P*-value < 0.10 for four root phenotypes: three genes (*LOC_Os01g04630*, *LOC_Os03g31480*, and *LOC_Os03g02750*) for RD_C, one gene (*LOC_Os02g54580*) for RV_C and RDW, and one gene (*LOC_Os12g32536*) for RD_L. Among the five genes, only *LOC_Os03g31480* (*OsEXPA31*) and *LOC_Os03g02750* (*OsSub25*) had a gene symbol in RAP-DB (Additional File 1; Table S5).

In addition to these significant associations, we focused on the genes with the top 10 strongest associations for each phenotype to identify other candidate genes. Since some genes were repeatedly detected in the top 10 associations for multiple phenotypes, 70 unique genes were involved in the 120 gene–phenotype associations (Additional File 1; Table S6). Candidate genes were screened based on their annotation information and correlation with the associated phenotype. We found that 31/70 genes had at least one gene symbol in RAP-DB, and 24/31 genes showed a moderate or strong correlation (absolute Pearson’s correlation > 0.50) with at least one phenotype in the 57 WRC accessions. Based on previous studies of these 24 genes, we selected five candidate genes possibly associated with root phenotypes (Table 2).

**Table 2:** Summary of the five genes selected from the top 10 associations in TWAS

Phenotype	Phenotype abbreviation	MSU ID	Gene Symbol	Functional description	TWAS rank	−log*P*	FDR adjusted *P*-value	Pearson’s correlation between the expression profile and the phenotype				
								All WRC (*n* = 57)	admixed (*n* = 7)	*aus* (*n* = 19)	*indica* (*n* = 21)	*japonica* (*n* = 10)
Crown root diameter	RD_C	*LOC_Os03g31480*	*OsEXPA31*	α-expansin subfamily, mediating cell wall loosening	1	5.15	0.06	−0.69	−0.38	−0.76	−0.60	−0.80
Crown root diameter	RD_C	*LOC_Os08g39890*	*OsSPL14*	Transcription activator/SPL14, a master regulator of rice plant architecture	5	4.32	0.15	0.76	0.70	0.87	0.74	0.72
The ratio of deep rooting	RDR	*LOC_Os09g26999*	*OsDEP1*	*Dense and erect panicle 1*, a pleiotropic gene for grain yield	5	4.08	0.28	0.54	0.39	0.20	0.68	0.21
The number of lateral root tips	NRT_L	*LOC_Os08g10450*	*OsENT1*	Equilibrative nucleoside transporter 1	6	3.88	0.37	−0.69	−0.14	−0.64	−0.47	−0.28
Crown root length	RL_C	*LOC_Os12g42250*	*OsDEC1*	*Decelerator of internode elongation 1*, encoding a zinc-finger transcription factor	9	3.58	0.34	−0.64	−0.43	−0.64	−0.43	−0.50

TWAS; transcriptome-wide association study, WRC; World Rice Core Collection

*OsENT1* was one of the five candidates responsible for NRT_L, which was also a candidate gene according to the GWAS. The other candidate genes according to TWAS (*OsEXPA31*, *OsSPL14*, *OsDEP1*, and *OsDEC1*) were not candidate genes according to the GWAS, but listed as promising candidates according to the results of previous studies. The first candidate gene, *OsEXPA31*, had the strongest association with RD_C and was one of the six significant (FDR-adjusted *P*-value < 0.10) candidate genes according to TWAS. Although *OsEXPA31* has not been functionally characterized, other α-expansin genes have been characterized for their involvement in root phenotypes, such as primary root length or root hair elongation (Ma et al. 2013; Wang et al. 2014b; Che et al. 2016; Yu et al. 2011). The second candidate gene *OsSPL14* was positively associated with RD_C in the WRC accessions (*r* = 0.76; −log*P* = 4.32) as well as in each subpopulation (*r* = 0.70–0.87), which was consistent with the results of a recent mutant-line-based study reporting that the crown roots thickened after increasing *OsSPL14* expression in the roots (Song et al. 2022). The third candidate gene *OsDEP1* has pleiotropic effects, including primary root elongation under limited phosphorus conditions (Sun et al. 2014; Zhang et al. 2015; Wang et al. 2021), and the positive association between *OsDEP1* and RDR (*r* = 0.54; −log*P* = 4.08) found in this study implies an uninvestigated function of the gene for the root architecture. Lastly, *OsDEC1* was negatively associated with RL_C (*r* = − 0.64 in the 57 WRC accessions), which seems to be in line with the negative effect of *OsDEC1* on internode elongation (Gómez-Ariza et al. 2019; Nagai et al. 2020).

### The GO Enrichment Analysis Highlighted Two Biological Processes Responsible for RD_C

If a biological process is related to a root phenotype, the genes involved in that biological process tend to show a strong association with the phenotype in the TWAS. Thus, we applied GO enrichment analysis to the genes with the top 1% positive and negative associations detected in TWAS to identify the biological processes related to the genetic variation of root phenotypes (Additional File 2; Figure S3). When the top 1% positive or negative associations were compared among the 12 root phenotypes, they highly overlapped among the four root-size-related phenotypes (RL, RSA, RV, and NRT) for both crown and lateral roots (Additional File 2; Figure S8). The average overlap rate was 69.5%, ranging from 31.4% (53/169 genes overlapped between the top 1% negative associations for RL_C and RV_C) to 94.1% (159/169 genes overlapped between the top 1% positive associations for RL_L and RSA_L). Therefore, we merged the top 1% associations for the four root size-related phenotypes (RS_C and RS_L for crown and lateral roots, respectively) for enrichment analysis to simplify interpretation. After combining the top 1% associations for the four phenotypes, 303 and 227 genes were positively associated with RS_C and RS_L, respectively, while 320 and 233 genes were negatively associated with RS_C and RS_L, respectively.

GO enrichment analysis identified 11 biological processes involved in the five root phenotypes (Table 3). While only one biological process related to RS_C, RS_L, and RDR, four biological processes related to RD_C and RD_L. Particularly, the top 1% genes negatively associated with RD_C were enriched in two explicable biological processes (GO:0009664 and GO:0006979). GO:0009664 was annotated to “plant-type cell wall organization” and assigned to five genes encoding α-expansin: *OsEXPA3* (*LOC_Os05g19570*), *OsEXPA9* (*LOC_Os01g14660*), *OsEXPA18* (*LOC_Os03g06040*), *OsEXPA19* (*LOC_Os03g06050*), and *OsEXPA31* (*LOC_Os03g31480*). This enrichment is consistent with the known role of expansins in root growth by mediating cell wall loosening (Zhang et al. 2021). GO:0006979 was annotated to “response to oxidative stress” and assigned to six genes encoding peroxidases: *OsPOD* (*LOC_Os01g19020*), *OsPRX42* (*LOC_Os03g25280*), *OsPRX43* (*LOC_Os03g25300*), *OsPRX54* (*LOC_Os04g34630*), *OsPRX68* (*LOC_Os05g04450*), and *OsPRX102* (*LOC_Os07g31610*). Although peroxidases are involved in several physiological processes throughout the plant life cycle, one of their major roles is cell wall modification and loosening by regulating the reactive oxygen species level (Passardi et al. 2004). Collectively, our GO enrichment results imply a potential physiological function of expansins and peroxidases in root diameter by regulating cell wall loosening.

**Table 3:** The 11 enriched biological processes in the GO enrichment analysis

Phenotype	Phenotype abbreviation	Direction of the association	GO ID	Description	# of genes with the tested annotation in the top 1% gene set	# of genes with an annotation in the top 1% gene set	# of genes with the tested annotation in the entire gene set^a^	# of genes with an annotation in the entire gene set^a^	*P*-value	FDR-adjusted *P*-value
Crown root size	RS_C	Positive	GO:0006260	DNA replication	8	117	51	6051	4.89 × 10^− 6^	2.44 × 10^− 4^
Lateral root size	RS_L	Positive	GO:0006412	Translation	24	89	276	6051	5.49 × 10^− 13^	2.42 × 10^− 11^
Crown root diameter	RD_C	Positive	GO:0043086	Negative regulation of catalytic activity	3	69	16	6051	7.15 × 10^− 4^	2.50 × 10^− 2^
Crown root diameter	RD_C	Positive	GO:0006096	Glycolysis	4	69	45	6051	1.62 × 10^− 3^	2.84 × 10^− 2^
Crown root diameter	RD_C	Negative	GO:0009664	Plant-type cell wall organization	5	69	28	6051	1.34 × 10^− 5^	3.74 × 10^− 4^
Crown root diameter	RD_C	Negative	GO:0006979	Response to oxidative stress	6	69	70	6051	1.30 × 10^− 4^	1.82 × 10^− 3^
Lateral root diameter	RD_L	Negative	GO:0006457	Protein folding	10	56	139	6051	4.28 × 10^− 7^	9.41 × 10^− 6^
Lateral root diameter	RD_L	Negative	GO:0009408	Response to heat	3	56	10	6051	8.61 × 10^− 5^	9.47 × 10^− 4^
Lateral root diameter	RD_L	Negative	GO:0019538	Protein metabolic process	3	56	12	6051	1.56 × 10^− 4^	1.14 × 10^− 3^
Lateral root diameter	RD_L	Negative	GO:0006950	Response to stress	3	56	33	6051	3.37 × 10^− 3^	1.85 × 10^− 2^
The ratio of deep rooting	RDR	Positive	GO:0006310	DNA recombination	3	69	14	6051	4.72 × 10^− 4^	1.79 × 10^− 2^

GO; gene ontology, FDR; false discovery rate

^a^The entire gene set consisted of 16,901 genes expressed in the root. As some genes were not annotated and some gene IDs were not convertible from MSU to RAP, the number of background genes reduced to 6,051 in the analysis

### eGWAS Discovered Two Overlaps between GWAS and TWAS

**Fig. 3:**
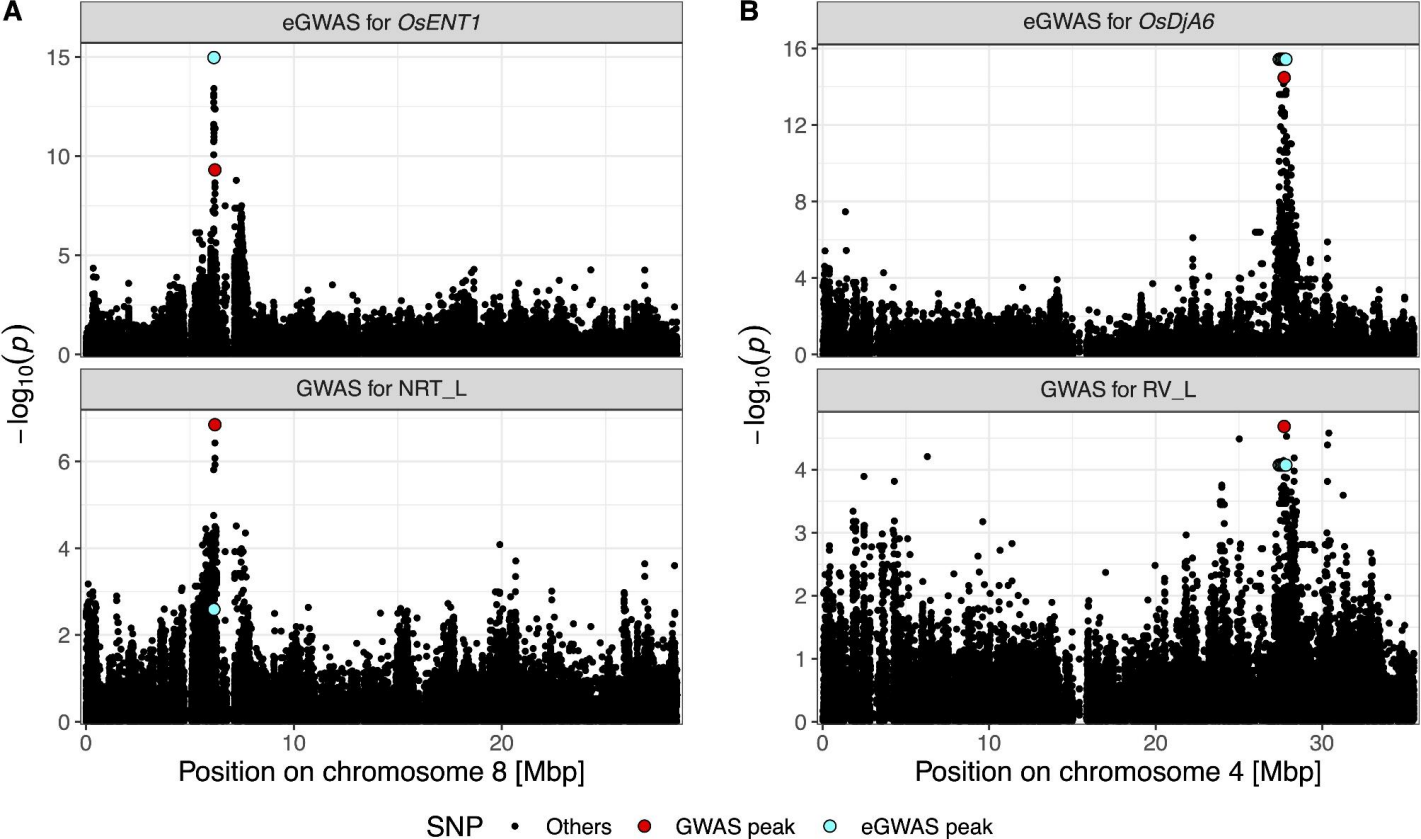
Two overlaps between the eGWAS and GWAS peaks. The chromosome-level Manhattan plots were visualized for the (**A**) eGWAS for *OsENT1* and GWAS for NRT_L on chromosome 8 and (**B**) eGWAS for *OsDjA6* and GWAS for RV_L on chromosome 4. The most significant GWAS SNP on the illustrated chromosome was highlighted in larger red dot than the other SNPs. Similarly, the eGWAS peak SNP was highlighted in larger cyan dot than the other SNPs

We applied eGWAS to the 70 candidate genes in the TWAS (Additional File 1; Table S6) to identify a strong *cis*- or *trans*-effect variant of the candidate gene, which enabled us to connect the results from the TWAS and GWAS (Additional File 2; Figure S3). None of the 70 TWAS candidate genes tested in the eGWAS showed a significant *trans*-eGWAS peak SNP, probably because of the smaller sizes of the *trans*-effects than that of the *cis*-effects (Wang et al. 2010, 2014a; Liu et al. 2022). Significant *cis*-eGWAS peaks were identified for six of the 70 TWAS candidate genes, which comprised 10 gene–phenotype associations (Additional File 1: Table S7). Two combinations (*OsENT1* responsible for NRT_L and *OsDjA6* responsible for RV_L) were eventually identified as common associations detected in all three statistical mapping methods (Fig. 3; Additional File 1: Table S7).

As expected from the GWAS and TWAS results, one of the two common associations was that between *OsENT1* and NRT_L. The eGWAS peak SNP was detected approximately 9 kb upstream of *OsENT1* with a highly significant signal (− log*P* = 14.97) and was located within 50 kb of the GWAS peak SNP responsible for NRT_L (Fig. 3A). We found that 30 of the 43 variants located 2 kb upstream of the end of the *OsENT1* region showed high LD (*r*^2^ > 0.90) with the eGWAS peak SNP (Additional File 1: Table S8). In particular, a SNP variant at a putative splicing site (7 bp downstream from the first exon) was in almost perfect LD with the eGWAS peak SNP (*r*^2^ = 0.96) and showed a visible relationship with *OsENT1* expression profile as well as with NRT_L phenotypic values (Additional File 2: Figure S9). Altogether, our results suggest that *OsENT1* is the most promising candidate gene responsible for NRT_L, although further experimental validation is required as the pairwise LD between the eGWAS and GWAS peak SNPs was moderate (*r*^2^ = 0.56).

The other common association was observed between *OsDjA6* (*LOC_Os04g46390*) and RV_L (Fig. 3B). Five equally significant *cis*-eGWAS peak SNPs were detected for *OsDjA6* from 27,425,399 to 27,820,992 bp on chromosome 4, covering the region of this gene (Additional File 1: Table S7). When the GWAS results for RV_L were compared with the eGWAS results, the most significant GWAS peak SNP at 27,719,033 bp (200 kb downstream of *OsDjA6* but between the two eGWAS peak SNPs at 27,625,119 and 27,725,503 bp) on chromosome 4 was in high LD with eGWAS peak SNPs (*r*^2^ = 0.97). Moreover, 24 polymorphic variants were present from 2 kb upstream to the end of the *OsDjA6* gene region in the 57 WRC accessions, of which nine variants showed high LD (*r*^2^ > 0.90) with both eGWAS and GWAS peak SNPs (Additional File 1: Table S9). Among these high-LD variants, two were in an exon but expected to be synonymous variants, three were in an intron, and four were upstream of *OsDjA6.* We then investigated the four upstream high-LD variants using the PLACE database (Higo et al. 1999) visualized in the JBrowse of RAP-DB, and discovered a SNP variant from adenine (reference allele) to guanine (alternative allele) at 27,504,969 bp (518 bp upstream of the representative MSU7 gene model of *OsDjA6*) located in a TATA box-like motif, whereas the other three were not located in any putative promoter motif. This SNP exhibited the same segregation pattern to the eGWAS peak SNP in the 57 WRC accessions: eight *indica* accessions (WRC03, WRC05, WRC07, WRC10, WRC12, WRC13, WRC16, and WRC19) had alternative alleles, whereas the remaining 49 accessions had reference alleles (Additional File 1: Table S10). These eight *indica* accessions showed a low *OsDjA6* expression profile, probably because of TATA-like motif mutation, which may explain their higher RV_L values than that of the other 13 *indica* accessions (Additional File 2: Figure S10). Thus, this *cis*-variant in the putative promoter motif is the most plausible source of the negative relationship between *OsDjA6* expression profiles and RV_L in *indica* subpopulation. Collectively, all GWAS, TWAS, and eGWAS supported the association between *OsDjA6* and RV_L.

## Discussion

To optimize the root system architecture in rice through molecular breeding, statistical mapping is a promising approach for identifying the candidate genes by leveraging the natural variation in root phenotypes. Thus, we used GWAS, TWAS, and eGWAS to explore the candidate genes related to the natural variation of the 12 root phenotypes using the genotypes of 424,888 SNPs and the expression profiles of 16,901 genes in 57 rice accessions. Our comprehensive statistical analyses identified *OsENT1*, *OsEXPA31*, *OsDEC1*, *OsSPL14*, *OsDEP1*, and *OsDjA6* as the candidate genes for root phenotypes. Furthermore, four significant genes (*LOC_Os01g04630*, *LOC_Os03g02750*, *LOC_Os02g54580*, and *LOC_Os12g32536*) and two weakly significant (FDR-adjusted *P*-value between 5% and 10%) genomic regions associated with at least one root phenotype were identified using TWAS and GWAS, respectively. In addition, GO enrichment analysis highlighted the importance of genes related to cell wall organization and response to oxidative stress in the natural variation in RD_C. While the sample size (*n* = 57) was limited to detect small-effect genes or loci, our statistical analyses dissected the genetic control of root phenotypes in a diverse panel and identified the candidate genes for molecular breeding and functional genomics of root system architecture.

All statistical analyses suggested that *OsENT1* is a candidate gene responsible for NRT_L. Both GWAS and eGWAS identified a significant peak in proximity to this gene, with the sixth strongest association for NRT_L in TWAS, and there was a SNP variant at a putative splicing site showing a high LD with the *cis*-eGWAS peak SNP. Based on the RiceXPro database (Sato et al. 2011b, 2013), *OsENT1* (RiceXPro accession ID: AK059439) expression profiles in roots, particularly in the root elongation zone (RXP_5002; Takehisa et al. 2013), were stronger than that in other organs (RiceXPro dataset ID: RXP_0001; Sato et al. 2011a) and exhibited a positive response to cytokinins in both roots (RXP_1005) and the shoots (RXP_1010). Four genes (*OsENT1–4*) encoding potential equilibrative nucleoside transporters have been identified in rice. Although the function of *OsENT1* in rice is not well understood, an expression analysis in yeast cells suggested that *OsENT2* plays a role as a cytokinin transporter (Hirose et al. 2005). Additionally, two equilibrative nucleoside transporter homolog genes in *Arabidopsis*, *AtENT3* and *AtENT8*, are involved in nucleoside-type cytokinin transport (Sun et al. 2005). While *OsENT1* did not show ability to transport nucleoside-type cytokinins, its amino acid sequence was the most homologous (45%) with that of *AtENT8* (Hirose et al. 2005). Cytokinins control the cell differentiation rate in the root meristem and, therefore, control root meristem size, crown root, and lateral root formation (Beemster and Baskin 2000; Dello Ioio et al. 2007; Laplaze et al. 2007; Neogy et al. 2021). Thus, we hypothesized that *OsENT1* may participate in cytokinin transport, thus affecting root phenotypes.

TWAS and GO enrichment analyses revealed that five α-expansin genes (*OsEXPA3*, *OsEXPA9*, *OsEXPA18*, *OsEXPA19*, and *OsEXPA31*) were negatively associated with RD_C, of which *OsEXPA31* showed the strongest association and a strong negative correlation with RD_C (*r* = − 0.69) in the 57 WRC accessions. The expansin proteins, discovered by McQueen-Mason et al. (1992), are a class of cell-wall-loosening proteins that play important roles in mediating plant growth and development (Cosgrove et al. 2002). Although *OsEXPA31* has not yet been functionally characterized, the relationships between other *OsEXPA*s and root morphology have been investigated in rice. *OsEXPA8* positively regulates primary root length and the number of lateral roots by mediating cell wall loosening (Ma et al. 2013; Wang et al. 2014b). *OsEXPA10* is required for root cell elongation (Che et al. 2016). *OsEXPA17* and *OsEXPA30* are root hair-specific genes that play crucial roles in root hair elongation (Yu et al. 2011). Interestingly, the protein sequence of OsEXPA31 is largely different from the four above expansins (He et al. 2015), implying a potential functional divergence of *OsEXPA31*. Therefore, our results suggest an undiscovered role of *OsEXPA31* for crown root diameter by regulating cell wall structure.

Most genes with the strongest association with root phenotypes in the TWAS were uncharacterized in rice, whereas three candidate genes had been reported to affect aboveground phenotypes. For instance, *OsDEC1*, which decelerates internode elongation, was negatively associated with RL_C in our study (*r* = − 0.64; Gómez-Ariza et al. 2019; Nagai et al. 2020). The well-known yield-related gene, *OsSPL14* showed the fifth strongest association with RD_C in the TWAS. Several studies have demonstrated that *OsSPL14* optimizes rice plant architecture and improves abiotic stress tolerance (Miura et al. 2010; Jiao et al. 2010; Zhu et al. 2022). *OsSPL14* also confers root elongation by modulating *PIN2* and *PIN10b* (auxin efflux carriers) transcription under low nitrogen supply (Wang et al. 2022). In a recent study, the crown root diameter was enlarged in *OsSPL14*-promoter-mutant plants that highly expressed this gene specifically in roots (Song et al. 2022), which is consistent with our association detected in TWAS. Another yield-related gene, *OsDEP1*, which is positively regulated by *OsSPL14* (Lu et al. 2013), was associated with RDR in the WRC57 accessions. *OsDEP1* was first reported to mediate panicle morphology and contribute to grain number improvement (Zhou et al. 2009; Huang et al. 2009), and further studies have demonstrated that it regulates nitrogen use efficiency and drought adaptation (Sun et al. 2014; Zhang et al. 2015). In addition, *OsDEP1* modulates root elongation for phosphorus uptake in rice (Wang et al. 2021). Considering the multiple functions of *OsDEP1* in plant growth and development (Trusov et al. 2007; Wang et al. 2006; Xu et al. 2016), *OsDEP1* may have undiscovered pleiotropic roles in other crucial growth processes in rice. Our results imply that yield-related genes such as *OsSPL14* and *OsDEP1* improve yield by modulating both shoot and root phenotypes.

Our comprehensive GWAS, TWAS, and eGWAS analyses identified a negative association between the expression profile of *OsDjA6* and RV_L. *OsDjA6* was characterized as a negative regulator of rice immunity to the blast fungus by regulating the genes involved in salicylic acid pathway, including the transcription factor *OsWRKY45* (Zhong et al. 2018). Considering the complex *OsWRKY45* regulatory mechanism in balancing plant growth and immune responses (Shimono et al. 2007; Wang et al. 2018b; Ichimaru et al. 2022), the association between *OsDjA6* and RV_L may imply an unknown pleiotropic function of *OsDjA6* by negatively regulating *OsWRKY45*.

Our results highlight the advantage of transcriptomics for candidate gene search, as none of the six candidate genes (*OsENT1*, *OsEXPA31*, *OsSPL14*, *OsDEP1*, *OsDEC1*, and *OsDjA6*) could be identified without transcriptome data. Although *OsENT1* was detected within ± 250 kb region from the GWAS peak SNP, it was impossible to shed light on its involvement without testing the statistical relationship between the expression profile and phenotypic value. Similarly, TWAS and eGWAS revealed a weak association of GWAS around the *cis*-regulatory region of *OsDjA6*. Although a GWAS can identify a genomic region associated with phenotypic variations, resolving the GWAS peak to a single candidate gene is often difficult. In contrast, the gene-level associations from the TWAS enabled us to discover novel associations of genes previously characterized for a shoot phenotype, such as *OsSPL14*, *OsDEP1*, and *OsDEC1*, with the root phenotypes.

## Conclusion

Association mapping analyses based on both transcriptome and genome data from the 57 WRC accessions revealed six associations between the genes and root phenotype: *OsENT1* was associated with NRT_L, *OsEXPA31* and *OsSPL14* with RD_C, *OsDEP1* with RDR, *OsDEC1* with RL_C, and *OsDjA6* with RV_L. These genes are promising targets for molecular breeding and functional genomics to understand the complex genetic control of root system architecture in rice.

### Electronic Supplementary Material

Below is the link to the electronicsupplementary material.

**Supplementary Material 1**: **Table S1**. BIC values and the selected models for GWAS and TWAS. **Table S2**. Candidate genes for the GWAS peak identified at 6,199,732 bp on chromosome 8. **Table S3**. Candidate genes for the GWAS peak identified at 20,665,890 bp on chromosome 8. **Table S4**. Candidate genes for the GWAS peak identified at 17,902,506 bp on chromosome 11. **Table S5**. Summary of the top ten gene-phenotype associations for the 12 root phenotypes in TWAS. **Table S6**. Candidate gene selection from the 70 unique genes in the top-ten gene-phenotype associations in TWAS. **Table S7**. Summary of the six genes with a significant *cis*-eGWAS peak. **Table S8**. Summary of the filtering, GWAS, and eGWAS results for the 43 polymorphic variants from 2 kb upsteam to the end of the *OsENT1* gene region. **Table S9**. Summary of the filtering, GWAS, and eGWAS results for the 24 polymorphic variants from 2 kb upsteam to the end of the *OsDjA6* gene region. **Table S10**. SNP genotype of the 57 WRC acccessions at the putative TATA box-like motif, at the GWAS peak SNP, and at the eGWAS peak SNP

**Supplementary Material 2**: **Figure S1**. Diagnostic plot of the factor relevance in the PEER analysis. **Figure S2**. Quantile-quantile plot of TWAS with or without the P3D option. **Figure S3**. Overview of the analysis pipeline. **Figure S4**. Manhattan and quantile-quantile plots for the 12 root phenotypes. **Figure S5**. LD heatmaps around the GWAS peak SNPs. **Figure S6**. Regional Manhattan plots colored by pairwise LD (*r*^*2*^) with the peak SNP. **Figure S7**. Genome-wide LD decay. **Figure S8**. The number of overlapped genes between the top 1% associations for different root phenotypes. **Figure S9**. The dependence of (A) the expression profile of OsENT1 and (B) the number of lateral root tips on the SNP at a putative splicing site of *OsENT1*. **Figure S10**. The dependence of (A) the expression profile of *OsDjA6* and (B) the lateral root volume on the SNP in a TATA box-like motif at an upstream of *OsDjA6* in *indica* subpopulation

## Data Availability

All data used in the present analyses have been published previously. The raw sequence data for SNP and indel discovery were deposited in the DNA Data Bank of Japan Sequence Read Archive in a previous study (Tanaka et al. 2020). Transcriptome data are available from the Gene Expression Omnibus (GSE162313) and root phenotype data are available in the supplementary file of the same publication (Kawakatsu et al. 2021). All codes for data analysis are available in Figshare (10.6084/m9.figshare.24116232.v1).

## Abbreviations

ANOVA: Analysis of variance
BIC: Bayesian information criterion
eGWAS: Expression genome-wide association study
eQTL: Expression quantitative trait loci
FDR: False discovery rate
FPKM: Fragments per kilobase exon per million reads
GO: Gene ontology
GWAS: Genome-wide association study
Indel: Insertion/deletion variant
LD: Linkage disequilibrium
MAF: Minor allele frequency
NRT_C: Number of crown root tips
NRT_L: Number of lateral root tips
PEER: Probabilistic estimation of the expression residuals
QQ: Quantile-quantile
QTL: Quantitative trait loci
RD_C: Crown root diameter
RD_L: Lateral root diameter
RDR: Ratio of deep rooting
RDW: Root dry weight
RL_C: Crown root length
RL_L: Lateral root length
RS_C: Crown root size
RS_L: Lateral root size
RSA_C: Crown root surface area
RSA_L: Lateral root surface area
RV_C: Crown root volume
RV_L: Lateral root volume
SNP: Single nucleotide polymorphism
TWAS: Transcriptome-wide association study
WRC: World rice core collection

## References

[CR1] Ahmadi N, Audebert A, Bennett MJ, Bishopp A, de Oliveira AC, Courtois B, Diedhiou A, Diévart A, Gantet P, Ghesquière A, Guiderdoni E, Henry A, Inukai Y, Kochian L, Laplaze L, Lucas M, Luu DT, Manneh B, Mo XR, Muthurajan R, Périn C, Price A, Robin S, Sentenac H, Sine B, Uga Y, Véry AA, Wissuwa M, Wu P, Xu J (2014). The roots of future rice harvests. Rice.

[CR2] Anandan A, Panda S, Sabarinathan S, Travis AJ, Norton GJ, Price AH (2022). Genetic basis and network underlying synergistic roots and shoots biomass accumulation revealed by genome-wide association studies in rice. Sci Rep.

[CR3] Beemster GT, Baskin TI (2000). STUNTED PLANT 1 mediates effects of cytokinin, but not of auxin, on cell division and expansion in the root of *Arabidopsis*. Plant Physiol.

[CR4] Bettembourg M, Dardou A, Audebert A, Thomas E, Frouin J, Guiderdoni E, Ahmadi N, Perin C, Dievart A, Courtois B (2017). Genome-wide association mapping for root cone angle in rice. Rice.

[CR5] Biscarini F, Cozzi P, Casella L, Riccardi P, Vattari A, Orasen G, Perrini R, Tacconi G, Tondelli A, Biselli C, Cattivelli L, Spindel J, McCouch S, Abbruscato P, Valé G, Piffanelli P, Greco R (2016). Genome-wide association study for traits related to plant and grain morphology, and root architecture in temperate rice accessions. PLoS ONE.

[CR6] Campbell MT, Du Q, Liu K, Sharma S, Zhang C, Walia H (2020). Characterization of the transcriptional divergence between the subspecies of cultivated rice (*Oryza sativa*). BMC Genomics.

[CR7] Chang CC, Chow CC, Tellier LC, Vattikuti S, Purcell SM, Lee JJ (2015). Second-generation PLINK: rising to the challenge of larger and richer datasets. GigaScience.

[CR8] Che J, Yamaji N, Shen RF, Ma JF (2016). An Al-inducible expansin gene, OsEXPA10 is involved in root cell elongation of rice. Plant J.

[CR9] Cingolani P, Platts A, Wang LL, Coon M, Nguyen T, Wang L, Land SJ, Lu X, Ruden DM (2012). A program for annotating and predicting the effects of single nucleotide polymorphisms. SnpEff Fly.

[CR10] Clark RT, Famoso AN, Zhao K, Shaff JE, Craft EJ, Bustamante CD, McCouch SR, Aneshansley DJ, Kochian LV (2013). High-throughput two-dimensional root system phenotyping platform facilitates genetic analysis of root growth and development. Plant Cell Environ.

[CR11] Cosgrove DJ, Li LC, Cho HT, Hoffmann-Benning S, Moore RC, Blecker D (2002). The growing world of expansins. Plant Cell Physiol.

[CR12] Courtois B, Audebert A, Dardou A, Roques S, Ghneim-Herrera T, Droc G, Frouin J, Rouan L, Gozé E, Kilian A, Ahmadi N, Dingkuhn M (2013). Genome-wide association mapping of root traits in a japonica rice panel. PLoS ONE.

[CR13] Danecek P, Auton A, Abecasis G, Albers CA, Banks E, DePristo MA, Handsaker RE, Lunter G, Marth GT, Sherry ST, McVean G, Durbin R, 1000 Genomes Project Analysis Group (2011). The variant call format and VCFtools. Bioinformatics.

[CR14] Dello Ioio R, Linhares FS, Scacchi E, Casamitjana-Martinez E, Heidstra R, Costantino P, Sabatini S (2007). Cytokinins determine *Arabidopsis* root-meristem size by controlling cell differentiation. Curr Biol.

[CR15] Dobin A, Davis CA, Schlesinger F, Drenkow J, Zaleski C, Jha S, Batut P, Chaisson M, Gingeras TR (2013). STAR: ultrafast universal RNA-seq aligner. Bioinformatics.

[CR16] Endelman JB (2011). Ridge regression and other kernels for genomic selection with R package rrBLUP. Plant Genome.

[CR17] Ferguson JN, Fernandes SB, Monier B, Miller ND, Allen D, Dmitrieva A, Schmuker P, Lozano R, Valluru R, Buckler ES, Gore MA, Brown PJ, Spalding EP, Leakey ADB (2021). Machine learning-enabled phenotyping for GWAS and TWAS of WUE traits in 869 field-grown sorghum accessions. Plant Physiol.

[CR18] Gómez-Ariza J, Brambilla V, Vicentini G, Landini M, Cerise M, Carrera E, Shrestha R, Chiozzotto R, Galbiati F, Caporali E, López Díaz I, Fornara F (2019). A transcription factor coordinating internode elongation and photoperiodic signals in rice. Nat Plants.

[CR19] Gowda VRP, Henry A, Yamauchi A, Shashidhar HE, Serraj R (2011). Root biology and genetic improvement for drought avoidance in rice. Field Crops Res.

[CR20] Groen SC, Ćalić I, Joly-Lopez Z, Platts AE, Choi JY, Natividad M, Dorph K, Mauck WM, Bracken B, Cabral CLU, Kumar A, Torres RO, Satija R, Vergara G, Henry A, Franks SJ, Purugganan MD (2020). The strength and pattern of natural selection on gene expression in rice. Nature.

[CR21] Groen SC, Joly-Lopez Z, Platts AE, Natividad M, Fresquez Z, Mauck WM, Quintana MR, Cabral CLU, Torres RO, Satija R, Purugganan MD, Henry A (2022). Evolutionary systems biology reveals patterns of rice adaptation to drought-prone agro-ecosystems. Plant Cell.

[CR22] Hanlon MT, Vejchasarn P, Fonta JE, Schneider HM, McCouch SR, Brown KM (2023). Genome wide association analysis of root hair traits in rice reveals novel genomic regions controlling epidermal cell differentiation. BMC Plant Biol.

[CR23] Harper AL, Trick M, Higgins J, Fraser F, Clissold L, Wells R, Hattori C, Werner P, Bancroft I (2012). Associative transcriptomics of traits in the polyploid crop species *Brassica napus*. Nat Biotechnol.

[CR24] He X, Zeng J, Cao F, Ahmed IM, Zhang G, Vincze E, Wu F (2015). *HvEXPB7*, a novel β-expansin gene revealed by the root hair transcriptome of tibetan wild barley, improves root hair growth under drought stress. J Exp Bot.

[CR25] Hershberger J, Tanaka R, Wood JC, Kaczmar N, Wu D, Hamilton JP, DellaPenna D, Buell CR, Gore MA (2022). Transcriptome-wide association and prediction for carotenoids and tocochromanols in fresh sweet corn kernels. Plant Genome.

[CR26] Higo K, Ugawa Y, Iwamoto M, Korenaga T (1999). Plant cis-acting regulatory DNA elements (PLACE) database: 1999. Nucleic Acids Res.

[CR27] Hirose N, Makita N, Yamaya T, Sakakibara H (2005). Functional characterization and expression analysis of a gene, OsENT2, encoding an equilibrative nucleoside transporter in rice suggest a function in cytokinin transport. Plant Physiol.

[CR28] Hirsch CN, Foerster JM, Johnson JM, Sekhon RS, Muttoni G, Vaillancourt B, Peñagaricano F, Lindquist E, Pedraza MA, Barry K, de Leon N, Kaeppler SM, Buell CR (2014). Insights into the maize pan-genome and pan-transcriptome. Plant Cell.

[CR29] Horiuchi Y, Harushima Y, Fujisawa H, Mochizuki T, Fujita M, Ohyanagi H, Kurata N (2015). Global expression differences and tissue specific expression differences in rice evolution result in two contrasting types of differentially expressed genes. BMC Genomics.

[CR30] Huang X, Qian Q, Liu Z, Sun H, He S, Luo D, Xia G, Chu C, Li J, Fu X (2009). Natural variation at the DEP1 locus enhances grain yield in rice. Nat Genet.

[CR31] Ichimaru K, Yamaguchi K, Harada K, Nishio Y, Hori M, Ishikawa K, Inoue H, Shigeta S, Inoue K, Shimada K, Yoshimura S, Takeda T, Yamashita E, Fujiwara T, Nakagawa A, Kojima C, Kawasaki T (2022). Cooperative regulation of PBI1 and MAPKs controls WRKY45 transcription factor in rice immunity. Nat Commun.

[CR32] Jiao Y, Wang Y, Xue D, Wang J, Yan M, Liu G, Dong G, Zeng D, Lu Z, Zhu X, Qian Q, Li J (2010). Regulation of OsSPL14 by OsmiR156 defines ideal plant architecture in rice. Nat Genet.

[CR33] Kang HM, Zaitlen NA, Wade CM, Kirby A, Heckerman D, Daly MJ, Eskin E (2008). Efficient control of population structure in model organism association mapping. Genetics.

[CR34] Kashima M, Sakamoto RL, Saito H, Ohkubo S, Tezuka A, Deguchi A, Hashida Y, Kurita Y, Iwayama K, Adachi S, Nagano AJ (2021). Genomic basis of transcriptome dynamics in rice under field conditions. Plant Cell Physiol.

[CR35] Kawahara Y, de la Bastide M, Hamilton JP, Kanamori H, McCombie WR, Ouyang S, Schwartz DC, Tanaka T, Wu J, Zhou S, Childs KL, Davidson RM, Lin H, Quesada-Ocampo L, Vaillancourt B, Sakai H, Lee SS, Kim J, Numa H, Itoh T, Buell CR, Matsumoto T (2013). Improvement of the *Oryza sativa* nipponbare reference genome using next generation sequence and optical map data. Rice.

[CR36] Kawakatsu T, Teramoto S, Takayasu S, Maruyama N, Nishijima R, Kitomi Y, Uga Y (2021). The transcriptomic landscapes of rice cultivars with diverse root system architectures grown in upland field conditions. Plant J.

[CR38] Kitomi Y, Kanno N, Kawai S, Mizubayashi T, Fukuoka S, Uga Y (2015). QTLs underlying natural variation of root growth angle among rice cultivars with the same functional allele of DEEPER ROOTING 1. Rice.

[CR37] Kitomi Y, Hanzawa E, Kuya N, Inoue H, Hara N, Kawai S, Kanno N, Endo M, Sugimoto K, Yamazaki T, Sakamoto S, Sentoku N, Wu J, Kanno H, Mitsuda N, Toriyama K, Sato T, Uga Y (2020). Root angle modifications by the *DRO1* homolog improve rice yields in saline paddy fields. Proc Nat Acad Sci U S A.

[CR39] Kliebenstein D (2009). Quantitative genomics: analyzing intraspecific variation using global gene expression polymorphisms or eQTLs. Annu Rev Plant Biol.

[CR40] Kojima Y, Ebana K, Fukuoka S, Nagamine T, Kawase M (2005). Development of an RFLP-based rice diversity research set of germplasm. Breed Sci.

[CR41] Kremling KAG, Chen SY, Su MH, Lepak NK, Romay MC, Swarts KL, Lu F, Lorant A, Bradbury PJ, Buckler ES (2018). Dysregulation of expression correlates with rare-allele burden and fitness loss in maize. Nature.

[CR42] Kremling KAG, Diepenbrock CH, Gore MA, Buckler ES, Bandillo NB (2019) Transcriptome-wide association supplements genome-wide association in *Zea mays*. G3 (Bethesda) 9: 3023–3033 10.1534/g3.119.400549 10.1534/g3.119.400549 PMC6723120 31337639

[CR43] Kumagai M, Nishikawa D, Kawahara Y, Wakimoto H, Itoh R, Tabei N, Tanaka T, Itoh T (2019). TASUKE+: a web-based platform for exploring GWAS results and large-scale resequencing data. DNA Res.

[CR44] Kuroha T, Nagai K, Kurokawa Y, Nagamura Y, Kusano M, Yasui H, Ashikari M, Fukushima A (2017). eQTLs regulating transcript variations associated with rapid internode elongation in deepwater rice. Front Plant Sci.

[CR45] Laplaze L, Benkova E, Casimiro I, Maes L, Vanneste S, Swarup R, Weijers D, Calvo V, Parizot B, Herrera-Rodriguez MB, Offringa R, Graham N, Doumas P, Friml J, Bogusz D, Beeckman T, Bennett M (2007). Cytokinins act directly on lateral root founder cells to inhibit root initiation. Plant Cell.

[CR47] Li H, Durbin R (2009). Fast and accurate short read alignment with Burrows-Wheeler transform. Bioinformatics.

[CR48] Li J, Han Y, Liu L, Chen Y, Du Y, Zhang J, Sun H, Zhao Q (2015). qRT9, a quantitative trait locus controlling root thickness and root length in upland rice. J Exp Bot.

[CR46] Li D, Liu Q, Schnable PS (2021). TWAS results are complementary to and less affected by linkage disequilibrium than GWAS. Plant Physiol.

[CR49] Li X, Yu B, Wu Q, Min Q, Zeng R, Xie Z, Huang J (2021). OsMADS23 phosphorylated by SAPK9 confers drought and salt tolerance by regulating ABA biosynthesis in rice. PLoS Genet.

[CR50] Liao Y, Smyth GK, Shi W (2014). featureCounts: an efficient general purpose program for assigning sequence reads to genomic features. Bioinformatics.

[CR51] Lin HY, Liu Q, Li X, Yang J, Liu S, Huang Y, Scanlon MJ, Nettleton D, Schnable PS (2017). Substantial contribution of genetic variation in the expression of transcription factors to phenotypic variation revealed by eRD-GWAS. Genome Biol.

[CR52] Liu C, Zhu X, Zhang J, Shen M, Chen K, Fu X, Ma L, Liu X, Zhou C, Zhou DX, Wang G (2022). eQTLs play critical roles in regulating gene expression and identifying key regulators in rice. Plant Biotechnol J.

[CR53] Lou Q, Chen L, Mei H, Wei H, Feng F, Wang P, Xia H, Li T, Luo L (2015). Quantitative trait locus mapping of deep rooting by linkage and association analysis in rice. J Exp Bot.

[CR54] Lou Q, Chen L, Mei H, Xu K, Wei H, Feng F, Li T, Pang X, Shi C, Luo L, Zhong Y (2017) Root transcriptomic analysis revealing the importance of energy metabolism to the development of deep roots in rice (*Oryza sativa* L). Front Plant Sci 8. 10.3389/fpls.2017.01314 10.3389/fpls.2017.01314 PMC5526896 28798764

[CR56] Lu Z, Yu H, Xiong G, Wang J, Jiao Y, Liu G, Jing Y, Meng X, Hu X, Qian Q, Fu X, Wang Y, Li J (2013). Genome-wide binding analysis of the transcription activator IDEAL PLANT ARCHITECTURE1 reveals a complex network regulating rice plant architecture. Plant Cell.

[CR55] Lu G, Harper AL, Trick M, Morgan C, Fraser F, O’Neill C, Bancroft I (2014). Associative transcriptomics study dissects the genetic architecture of seed glucosinolate content in *Brassica napus*. DNA Res.

[CR57] Ma N, Wang Y, Qiu S, Kang Z, Che S, Wang G, Huang J (2013). Overexpression of OsEXPA8, a root-specific gene, improves rice growth and root system architecture by facilitating cell extension. PLoS ONE.

[CR58] Mai CD, Phung NT, To HT, Gonin M, Hoang GT, Nguyen KL, Do VN, Courtois B, Gantet P (2014). Genes controlling root development in rice. Rice.

[CR59] McQueen-Mason S, Durachko DM, Cosgrove DJ (1992). Two endogenous proteins that induce cell wall extension in plants. Plant Cell.

[CR61] Meng F, Xiang D, Zhu J, Li Y, Mao C (2019). Molecular mechanisms of root development in rice. Rice.

[CR60] Miura K, Ikeda M, Matsubara A, Song XJ, Ito M, Asano K, Matsuoka M, Kitano H, Ashikari M (2010). OsSPL14 promotes panicle branching and higher grain productivity in rice. Nat Genet.

[CR62] Muthayya S, Sugimoto JD, Montgomery S, Maberly GF (2014). An overview of global rice production, supply, trade, and consumption. Ann NY Acad Sci.

[CR63] Nagai K, Mori Y, Ishikawa S, Furuta T, Gamuyao R, Niimi Y, Hobo T, Fukuda M, Kojima M, Takebayashi Y, Fukushima A, Himuro Y, Kobayashi M, Ackley W, Hisano H, Sato K, Yoshida A, Wu J, Sakakibara H, Sato Y, Tsuji H, Akagi T, Ashikari M (2020). Antagonistic regulation of the gibberellic acid response during stem growth in rice. Nature.

[CR64] Neogy A, Singh Z, Mushahary KKK, Yadav SR (2021). Dynamic cytokinin signaling and function of auxin in cytokinin responsive domains during rice crown root development. Plant Cell Rep.

[CR65] Passardi F, Penel C, Dunand C (2004). Performing the paradoxical: how plant peroxidases modify the cell wall. Trends Plant Sci.

[CR66] Phung NTP, Mai CD, Hoang GT, Truong HTM, Lavarenne J, Gonin M, Nguyen KL, Ha TT, Do VN, Gantet P, Courtois B (2016). Genome-wide association mapping for root traits in a panel of rice accessions from Vietnam. BMC Plant Biol.

[CR67] Pignon CP, Fernandes SB, Valluru R, Bandillo N, Lozano R, Buckler E, Gore MA, Long SP, Brown PJ, Leakey ADB (2021). Phenotyping stomatal closure by thermal imaging for GWAS and TWAS of water use efficiency-related genes. Plant Physiol.

[CR68] Purcell S, Neale B, Todd-Brown K, Thomas L, Ferreira MA, Bender D, Maller J, Sklar P, de Bakker PI, Daly MJ, Sham PC (2007). PLINK: a tool set for whole-genome association and population-based linkage analyses. Am J Hum Genet.

[CR69] Robinson MD, McCarthy DJ, Smyth GK (2010). edgeR: a Bioconductor package for differential expression analysis of digital gene expression data. Bioinformatics.

[CR70] Sakai H, Lee SS, Tanaka T, Numa H, Kim J, Kawahara Y, Wakimoto H, Yang CC, Iwamoto M, Abe T, Yamada Y, Muto A, Inokuchi H, Ikemura T, Matsumoto T, Sasaki T, Itoh T (2013). Rice annotation project database (RAP-DB): an integrative and interactive database for rice genomics. Plant Cell Physiol.

[CR71] Sato Y, Antonio B, Namiki N, Motoyama R, Sugimoto K, Takehisa H, Minami H, Kamatsuki K, Kusaba M, Hirochika H, Nagamura Y (2011). Field transcriptome revealed critical developmental and physiological transitions involved in the expression of growth potential in japonica rice. BMC Plant Biol.

[CR72] Sato Y, Antonio BA, Namiki N, Takehisa H, Minami H, Kamatsuki K, Sugimoto K, Shimizu Y, Hirochika H, Nagamura Y (2011). RiceXPro: a platform for monitoring gene expression in japonica rice grown under natural field conditions. Nucleic Acids Res.

[CR73] Sato Y, Takehisa H, Kamatsuki K, Minami H, Namiki N, Ikawa H, Ohyanagi H, Sugimoto K, Antonio BA, Nagamura Y (2013). RiceXPro Version 3.0: expanding the informatics resource for rice transcriptome. Nucleic Acids Res.

[CR74] Shimono M, Sugano S, Nakayama A, Jiang CJ, Ono K, Toki S, Takatsuji H (2007). Rice WRKY45 plays a crucial role in benzothiadiazole-inducible blast resistance. Plant Cell.

[CR75] Shin JH, Blay S, McNeney B, Graham J (2006). LDheatmap: an R function for graphical display of pairwise linkage disequilibria between single nucleotide polymorphisms. J Stat Softw.

[CR76] Song X, Meng X, Guo H, Cheng Q, Jing Y, Chen M, Liu G, Wang B, Wang Y, Li J, Yu H (2022). Targeting a gene regulatory element enhances rice grain yield by decoupling panicle number and size. Nat Biotechnol.

[CR79] Stegle O, Parts L, Piipari M, Winn J, Durbin R (2012). Using probabilistic estimation of expression residuals (PEER) to obtain increased power and interpretability of gene expression analyses. Nat Protoc.

[CR78] Sun J, Hirose N, Wang X, Wen P, Xue L, Sakakibara H, Zuo J (2005). Arabidopsis SOI33/AtENT8 gene encodes a putative equilibrative nucleoside transporter that is involved in cytokinin transport in planta. J Integr Plant Biol.

[CR77] Sun H, Qian Q, Wu K, Luo J, Wang S, Zhang C, Ma Y, Liu Q, Huang X, Yuan Q, Han R, Zhao M, Dong G, Guo L, Zhu X, Gou Z, Wang W, Wu Y, Lin H, Fu X (2014). Heterotrimeric G proteins regulate nitrogen-use efficiency in rice. Nat Genet.

[CR83] Takehisa H, Sato Y, Igarashi M, Abiko T, Antonio BA, Kamatsuki K, Minami H, Namiki N, Inukai Y, Nakazono M, Nagamura Y (2012). Genome-wide transcriptome dissection of the rice root system: implications for developmental and physiological functions. Plant J.

[CR82] Takehisa H, Sato Y, Antonio BA, Nagamura Y (2013). Global transcriptome profile of rice root in response to essential macronutrient deficiency. Plant Signal Behav.

[CR80] Tanaka N, Shenton M, Kawahara Y, Kumagai M, Sakai H, Kanamori H, Yonemaru J, Fukuoka S, Sugimoto K, Ishimoto M, Wu J, Ebana K (2020). Whole-genome sequencing of the NARO world rice core collection (WRC) as the basis for diversity and association studies. Plant Cell Physiol.

[CR81] Tang S, Zhao H, Lu S, Yu L, Zhang G, Zhang Y, Yang QY, Zhou Y, Wang X, Ma Wei, Xie W, Guo L (2021). Genome- and transcriptome-wide association studies provide insights into the genetic basis of natural variation of seed oil content in *Brassica napus*. Mol Plant.

[CR84] Teramoto S, Kitomi Y, Nishijima R, Takayasu S, Maruyama N, Uga Y (2019). Backhoe-assisted monolith method for plant root phenotyping under upland conditions. Breed Sci.

[CR85] Teramoto S, Yamasaki M, Uga Y (2022). Identification of a unique allele in the quantitative trait locus for crown root number in japonica rice from Japan using genome-wide association studies. Breed Sci.

[CR86] To HTM, Nguyen HT, Dang NTM, Nguyen NH, Bui TX, Lavarenne J, Phung NTP, Gantet P, Lebrun M, Bellafiore S, Champion A (2019). Unraveling the genetic elements involved in shoot and root growth regulation by jasmonate in rice using a genome-wide association study. Rice.

[CR87] Trusov Y, Rookes JE, Tilbrook K, Chakravorty D, Mason MG, Anderson D, Chen JG, Jones AM, Botella JR (2007). Heterotrimeric G protein γ subunits provide functional selectivity in Gβγ dimer signaling in *Arabidopsis*. Plant Cell.

[CR88] Turner SD (2018). Qqman: an R package for visualizing GWAS results using Q-Q and manhattan plots. J Open Source Softw.

[CR89] Uga Y, Ebana K, Abe J, Morita S, Okuno K, Yano M (2009). Variation in root morphology and anatomy among accessions of cultivated rice (*Oryza sativa* L.) with different genetic backgrounds. Breed Sci.

[CR91] Uga Y, Okuno K, Yano M (2011). Dro1, a major QTL involved in deep rooting of rice under upland field conditions. J Exp Bot.

[CR90] Uga Y, Hanzawa E, Nagai S, Sasaki K, Yano M, Sato T (2012). Identification of qSOR1, a major rice QTL involved in soil-surface rooting in paddy fields. Theor Appl Genet.

[CR92] Uga Y, Sugimoto K, Ogawa S, Rane J, Ishitani M, Hara N, Kitomi Y, Inukai Y, Ono K, Kanno N, Inoue H, Takehisa H, Motoyama R, Nagamura Y, Wu J, Matsumoto T, Takai T, Okuno K, Yano M (2013). Control of root system architecture by DEEPER ROOTING 1 increases rice yield under drought conditions. Nat Genet.

[CR93] Van der Auwera GA, Carneiro MO, Hartl C, Poplin R, Del Angel G, Levy-Moonshine A, Jordan T, Shakir K, Roazen D, Thibault J, Banks E, Garimella KV, Altshuler D, Gabriel S, DePristo MA (2013) From FastQ data to high confidence variant calls: the Genome Analysis Toolkit best practices pipeline. Curr Protoc Bioinformatics 43. 10.1002/0471250953.bi1110s43 10.1002/0471250953.bi1110s43 PMC4243306 25431634

[CR94] VanRaden PM (2008). Efficient methods to compute genomic predictions. J Dairy Sci.

[CR101] Wang L, Xu YY, Ma QB, Li D, Xu ZH, Chong K (2006). Heterotrimeric G protein α subunit is involved in rice brassinosteroid response. Cell Res.

[CR97] Wang J, Yu H, Xie W, Xing Y, Yu S, Xu C, Li X, Xiao J, Zhang Q (2010). A global analysis of QTLs for expression variations in rice shoots at the early seedling stage. Plant J.

[CR98] Wang J, Yu H, Weng X, Xie W, Xu C, Li X, Xiao J, Zhang Q (2014). An expression quantitative trait loci-guided co-expression analysis for constructing regulatory network using a rice recombinant inbred line population. J Exp Bot.

[CR103] Wang Y, Ma N, Qiu S, Zou H, Zhang G, Kang Z, Wang G, Huang J (2014). Regulation of the α-expansin gene OsEXPA8 expression affects root system architecture in transgenic rice plants. Mol Breed.

[CR96] Wang F, Longkumer T, Catausan SC, Calumpang CLF, Tarun JA, Cattin-Ortola J, Ishizaki T, Pariasca-Tanaka J, Rose T, Wissuwa M, Kretzschmar T (2018). Genome-wide association and gene validation studies for early root vigour to improve direct seeding of rice. Plant Cell Environ.

[CR99] Wang J, Zhou L, Shi H, Chern M, Yu H, Yi H, He M, Yin J, Zhu X, Li Y, Li W, Liu J, Wang J, Chen X, Qing H, Wang Y, Liu G, Wang W, Li P, Wu X, Zhu L, Zhou JM, Ronald PC, Li S, Li J, Chen X (2018). A single transcription factor promotes both yield and immunity in rice. Science.

[CR102] Wang X, Chen Q, Wu Y, Lemmon ZH, Xu G, Huang C, Liang Y, Xu D, Li D, Doebley JF, Tian F (2018). Genome-wide analysis of transcriptional variability in a large maize-teosinte population. Mol Plant.

[CR100] Wang K, Xu F, Yuan W, Zhang D, Liu J, Sun L, Cui L, Zhang J, Xu W (2021). Rice G protein γ subunit qPE9-1 modulates root elongation for phosphorus uptake by involving 14-3-3 protein OsGF14b and plasma membrane H^+^-ATPase. Plant J.

[CR95] Wang B, Guo X, Qi X, Feng F, Zhu X, Hu Y, Li J, Zhao Q, Sun H (2022). OsSPL14 is involved in nitrogen-deficiency-induced root elongation in rice. Environ Exp Bot.

[CR104] Wittkopp PJ, Haerum BK, Clark AG (2004). Evolutionary changes in cis and trans gene regulation. Nature.

[CR106] Wu T, Hu E, Xu S, Chen M, Guo P, Dai Z, Feng T, Zhou L, Tang W, Zhan L, Fu X, Liu S, Bo X, Yu G (2021). clusterProfiler 4.0: a universal enrichment tool for interpreting omics data. The Innovation.

[CR105] Wu D, Li X, Tanaka R, Wood JC, Tibbs-Cortes LE, Magallanes-Lundback M, Bornowski N, Hamilton JP, Vaillancourt Brieanne, Diepenbrock CH, Li X, Deason NT, Schoenbaum GR, Yu J, Buell CR, DellaPenna D, Gore MA (2022). Combining GWAS and TWAS to identify candidate causal genes for tocochromanol levels in maize grain. Genetics.

[CR107] Xiang J, Zhang C, Wang N, Liang Z, Zhenzhen Z, Liang L, Yuan H, Shi Y (2022). Genome-wide association study reveals candidate genes for root-related traits in rice. Curr Issues in Mol Biol.

[CR109] Xu H, Zhao M, Zhang Q, Xu Z, Xu Q (2016). The DENSE AND ERECT PANICLE 1 (DEP1) gene offering the potential in the breeding of high-yielding rice. Breed Sci.

[CR108] Xu X, Ye J, Yang Y, Zhang M, Xu Q, Feng Y, Yuan X, Yu H, Wang Y, Yang Y, Wei X (2020). Genome-wide association study of rice rooting ability at the seedling stage. Rice.

[CR110] Yoshino K, Nishijima R, Kawakatsu T (2020). Low-cost RNA extraction method for highly scalable transcriptome studies. Breed Sci.

[CR112] Yu Z, Kang B, He X, Lv S, Bai Y, Ding W, Chen M, Cho HT, Wu P (2011). Root hair-specific expansins modulate root hair elongation in rice. Plant J.

[CR111] Yu G, Wang LG, Han Y, He QY (2012). clusterProfiler: an R package for comparing biological themes among gene clusters. OMICS.

[CR115] Zhang DP, Zhou Y, Yin JF, Yan XJ, Lin S, Xu WF, Baluška F, Wang YP, Xia YJ, Liang GH, Liang JS (2015). Rice G-protein subunits qPE9-1 and RGB1 play distinct roles in abscisic acid responses and drought adaptation. J Exp Bot.

[CR117] Zhang L, Su W, Tao R, Zhang W, Chen J, Wu P, Yan C, Jia Y, Larkin RM, Lavelle D, Truco MJ, Chin-Wo SR, Michelmore RW, Kuang H (2017). RNA sequencing provides insights into the evolution of lettuce and the regulation of flavonoid biosynthesis. Nat Commun.

[CR118] Zhang W, Dai X, Xu S, Zhao PX (2018). 2D association and integrative omics analysis in rice provides systems biology view in trait analysis. Commun Biol.

[CR114] Zhang C, Dong SS, Xu JY, He WM, Yang TL (2019). PopLDdecay: a fast and effective tool for linkage disequilibrium decay analysis based on variant call format files. Bioinformatics.

[CR116] Zhang H, San ML, Jang S-G, Lee JH, Kim NE, Lee AR, Park SY, Cao FY, Chin JH, Kwon SW (2020). Genome-wide association study of root system development at seedling stage in rice. Genes.

[CR113] Zhang B, Gao Y, Zhang L, Zhou Y (2021). The plant cell wall: biosynthesis, construction, and functions. J Integr Plant Biol.

[CR121] Zhao Y, Zhang H, Xu J, Jiang C, Yin Z, Xiong H, Xie J, Wang X, Zhu X, Li Y, Zhao W, Rashid MAR, Li J, Wang W, Fu B, Ye G, Guo Y, Hu Z, Li Z, Li Z (2018). Loci and natural alleles underlying robust roots and adaptive domestication of upland ecotype rice in aerobic conditions. PLoS Genet.

[CR119] Zhao J, Yang B, Li W, Sun S, Peng L, Feng D, Li L, Di H, He Y, Wang Z (2021). A genome-wide association study reveals that the glucosyltransferase OsIAGLU regulates root growth in rice. J Exp Bot.

[CR120] Zhao Y, Yin Z, Wang X, Jiang C, Aslam MM, Gao F, Pan Y, Xie J, Zhu X, Dong L, Liu Y, Zhang H, Li J, Li Z (2021). Genetic basis and network underlying synergistic roots and shoots biomass accumulation revealed by genome-wide association studies in rice. Sci Rep.

[CR122] Zhong X, Yang J, Shi Y, Wang X, Wang GL (2018). The DnaJ protein OsDjA6 negatively regulates rice innate immunity to the blast fungus *Magnaporthe oryzae*. Mol Plant Pathol.

[CR123] Zhou Y, Zhu J, Li Z, Yi C, Liu J, Zhang H, Tang S, Gu M, Liang G (2009). Deletion in a quantitative trait gene qPE9-1 associated with panicle erectness improves plant architecture during rice domestication. Genetics.

[CR124] Zhu M, He Y, Zhu M, Ahmad A, Xu S, He Z, Jiang S, Huang J, Li Z, Liu S, Hou X, Zhang Z (2022). ipa1 improves rice drought tolerance at seedling stage mainly through activating abscisic acid pathway. Plant Cell Rep.

