# Supplementary material for: Genome- and Transcriptome-wide Association Studies to Discover Candidate Genes for Diverse Root Phenotypes in Cultivated Rice
Source: Rice (N Y). 2023 Dec 8;16:55. doi: 10.1186/s12284-023-00672-x (PMC10709265; doi:10.1186/s12284-023-00672-x)
Supplement: Supplementary file 2 — Supplementary Material 2: Figure S1. Diagnostic plot of the factor relevance in the PEER analysis. Figure S2. Quantile-quantile plot of TWAS with or without the P3D option. Figure S3. Overview of the analysis pipeline. Figure S4. Manhattan and quantile-quantile plots for the 12 root phenotypes. Figure S5. LD heatmaps around the GWAS peak SNPs. Figure S6. Regional Manhattan plots colored by pairwise LD (r2) with the peak SNP. Figure S7. Genome-wide LD decay. Figure S8. The number of overlapped genes between the top 1% associations for different root phenotypes. Figure S9. The dependence of (A) the expression profile of OsENT1 and (B) the number of lateral root tips on the SNP at a putative splicing site of OsENT1. Figure S10. The dependence of (A) the expression profile of OsDjA6 and (B) the lateral root volume on the SNP in a TATA box-like motif at an upstream of OsDjA6 in indica subpopulation [file 12284_2023_672_MOESM2_ESM.docx]

**Additional file 1: Supplemental Figures**


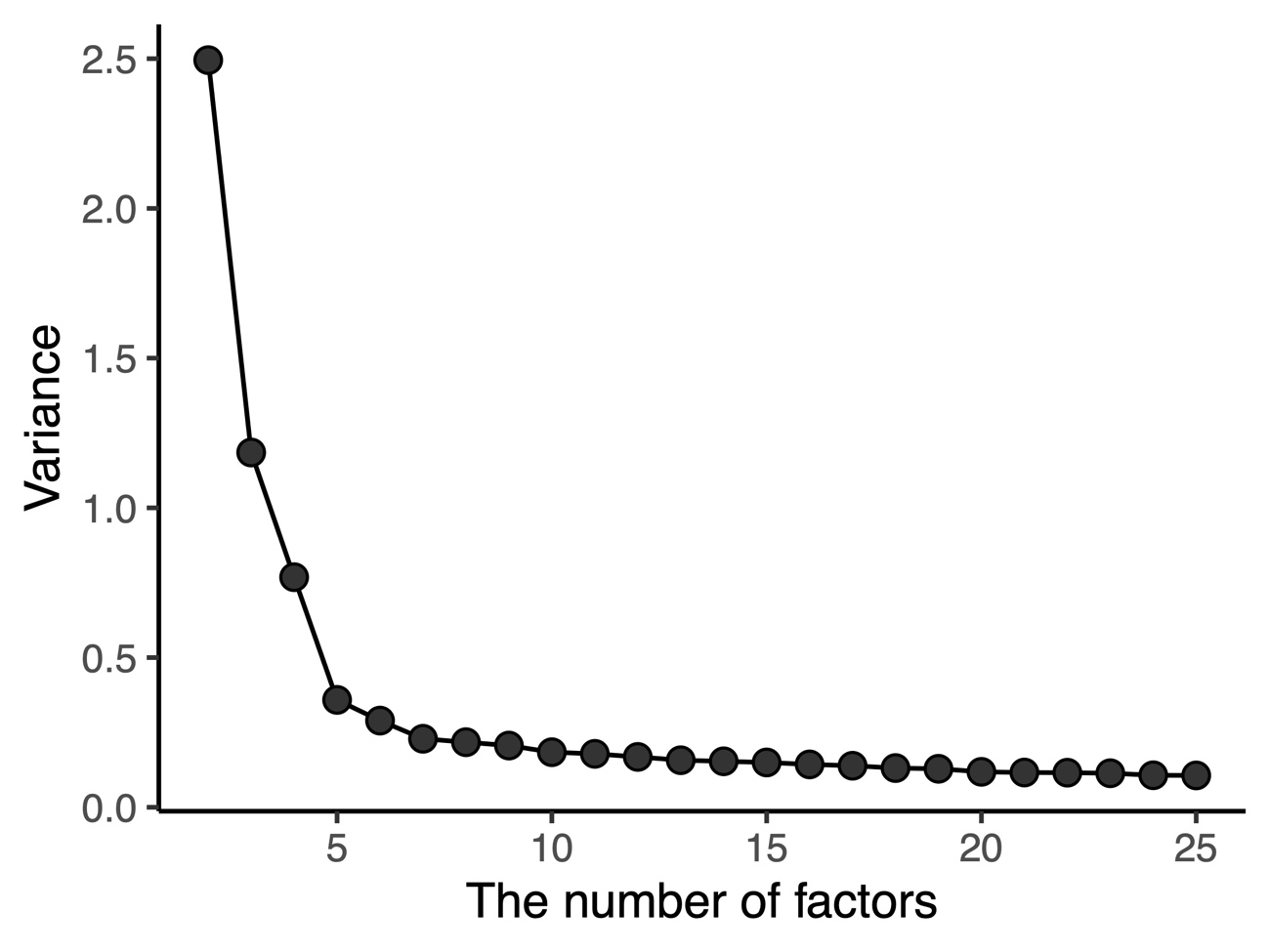


**Figure S1. Diagnostic plot of the factor relevance in the PEER analysis.** The optimal number of factors was set to five by visually identifying the “elbow” of the curve. The first factor was omitted from the figure, because it explained much larger variance (variance = 963.3) than the other factors and made the visual diagnosis difficult.

PEER; probabilistic estimation of the expression residuals


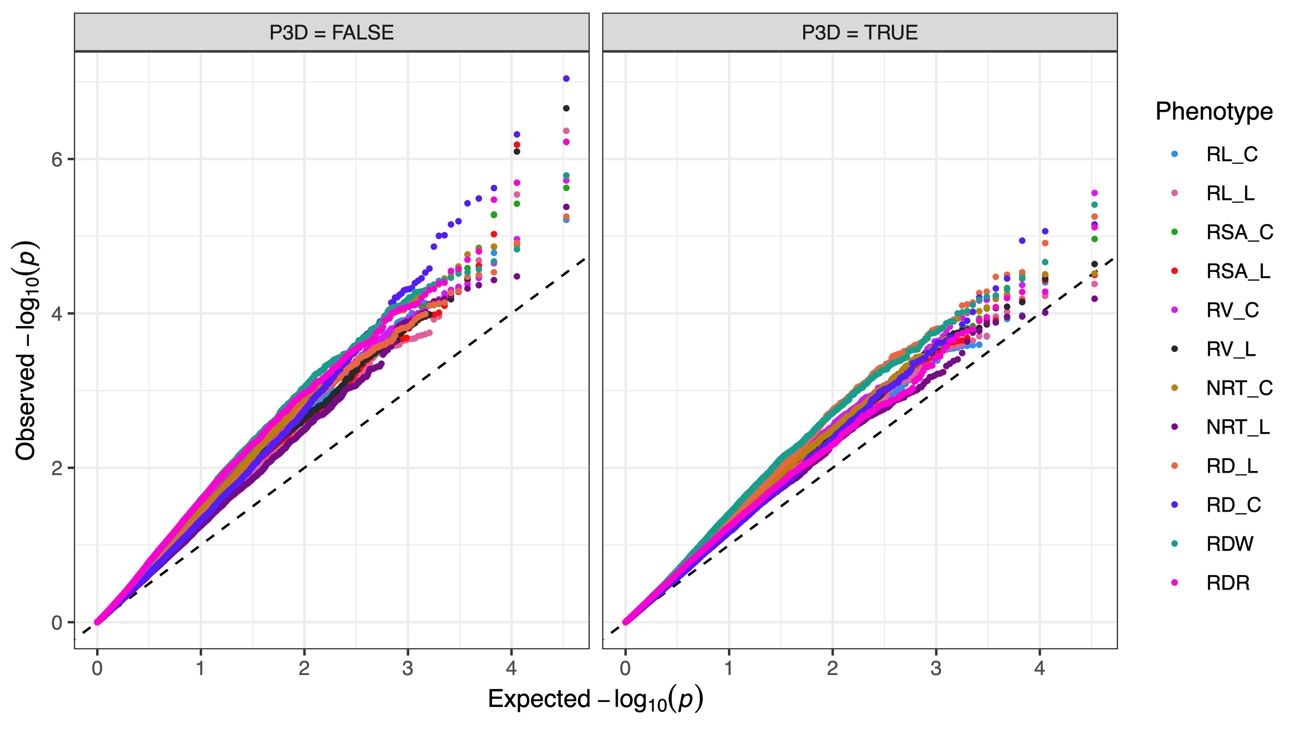


**Figure S2. Quantile-quantile plot of TWAS with or without the P3D option.** The inflation of *P*-values was found when the P3D option was set to FALSE (left panel), while the inflation was suppressed when the P3D option was set to TRUE (right panel). According to this visual diagnosis, P3D option was set to TRUE in TWAS.

TWAS; transcriptome-wide association study, RL_C; crown root length, RL_L; lateral root length, RSA_C; crown root surface area, RSA_L; lateral root surface area, RV_C; crown root volume, RV_L; lateral root volume, NRT_C; the number of crown root tips, NRT_L; the number of lateral root tips, RD_C; crown root diameter, RD_L; lateral root diameter, RDW; root dry weight, RDR; The ratio of deep rooting


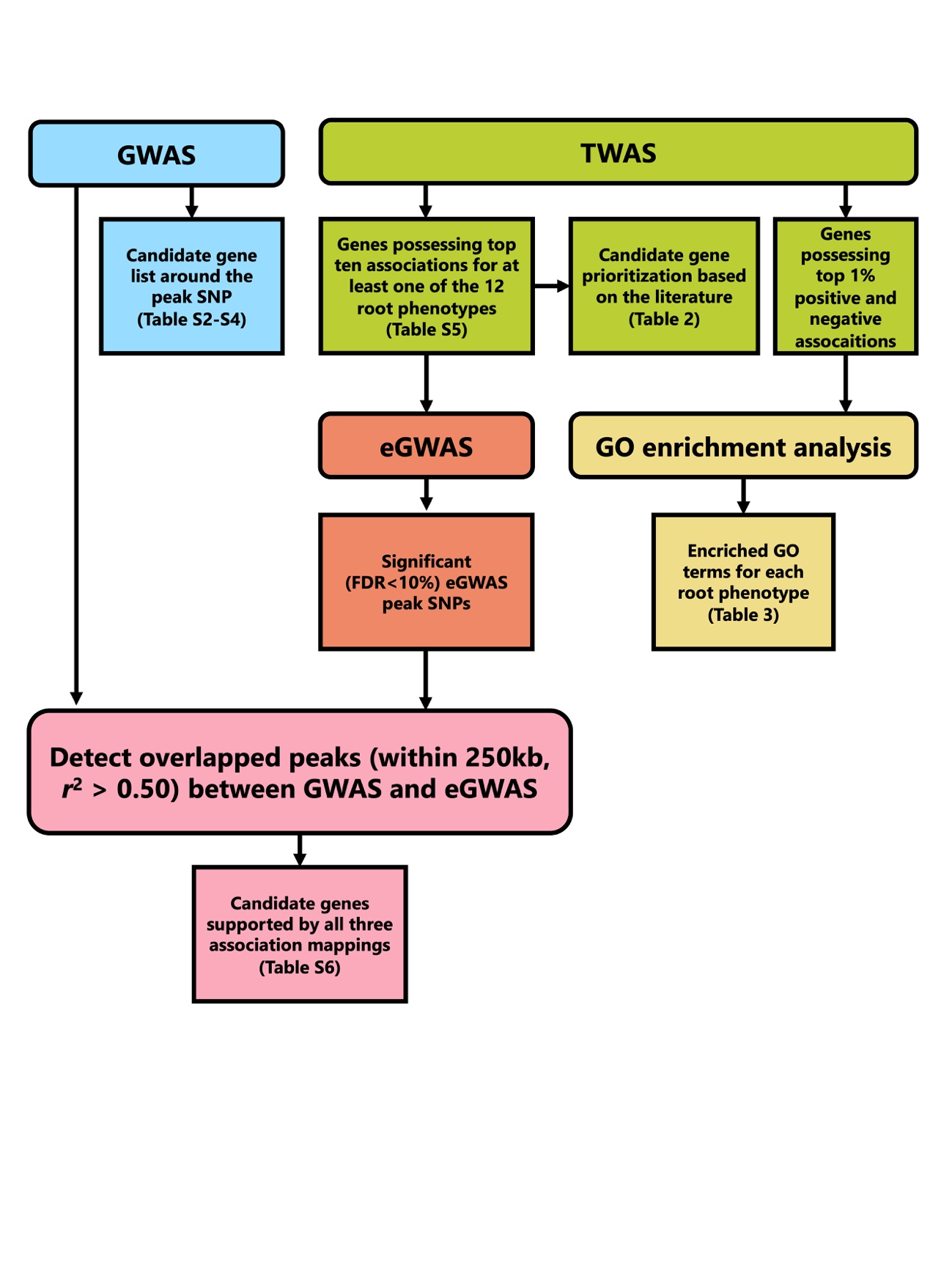


**Figure S3. Overview of the analysis pipeline.** Different colors represent different statistical methods (shown by the rounded rectangles) and relevant results (shown by the rectangles).

GWAS; genome-wide association study, TWAS; transcriptome-wide association study, SNP; single nucleotide polymorphism, eGWAS; expression genome-wide association study, GO; gene ontology, FDR; false discovery rate


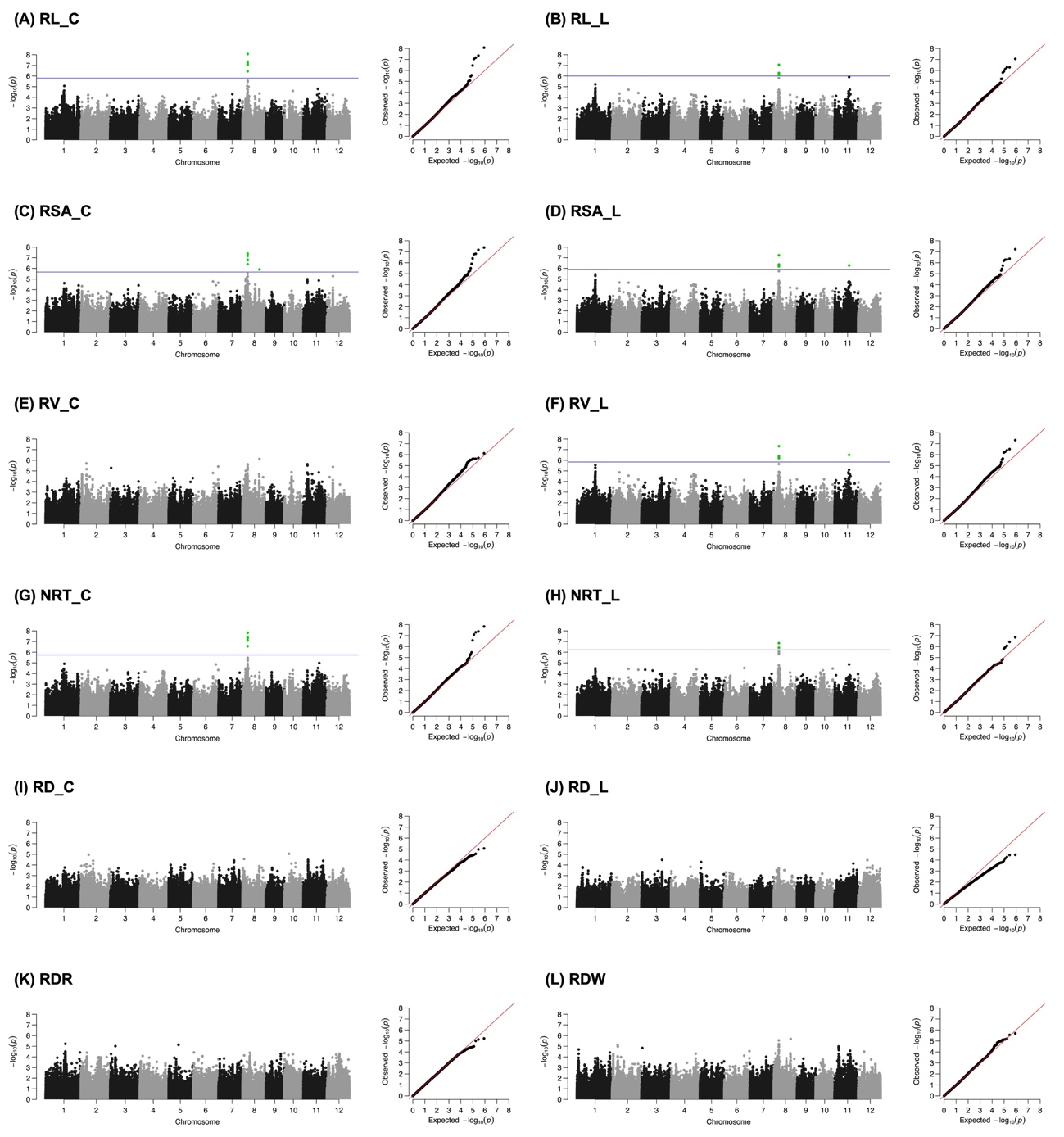


**Figure S4. Manhattan and quantile-quantile plots for the 12 root phenotypes.** The blue horizontal line in the Manhattan plot represents the FDR = 10% cutoff. The significant SNPs are highlighted by the green dots.

FDR; false-discovery rate, RL_C; crown root length, RL_L; lateral root length, RSA_C; crown root surface area, RSA_L; lateral root surface area, RV_C; crown root volume, RV_L; lateral root volume, NRT_C; the number of crown root tips, NRT_L; the number of lateral root tips, RD_C; crown root diameter, RD_L; lateral root diameter, RDR; ratio of deep rooting, RDW; root dry weight.


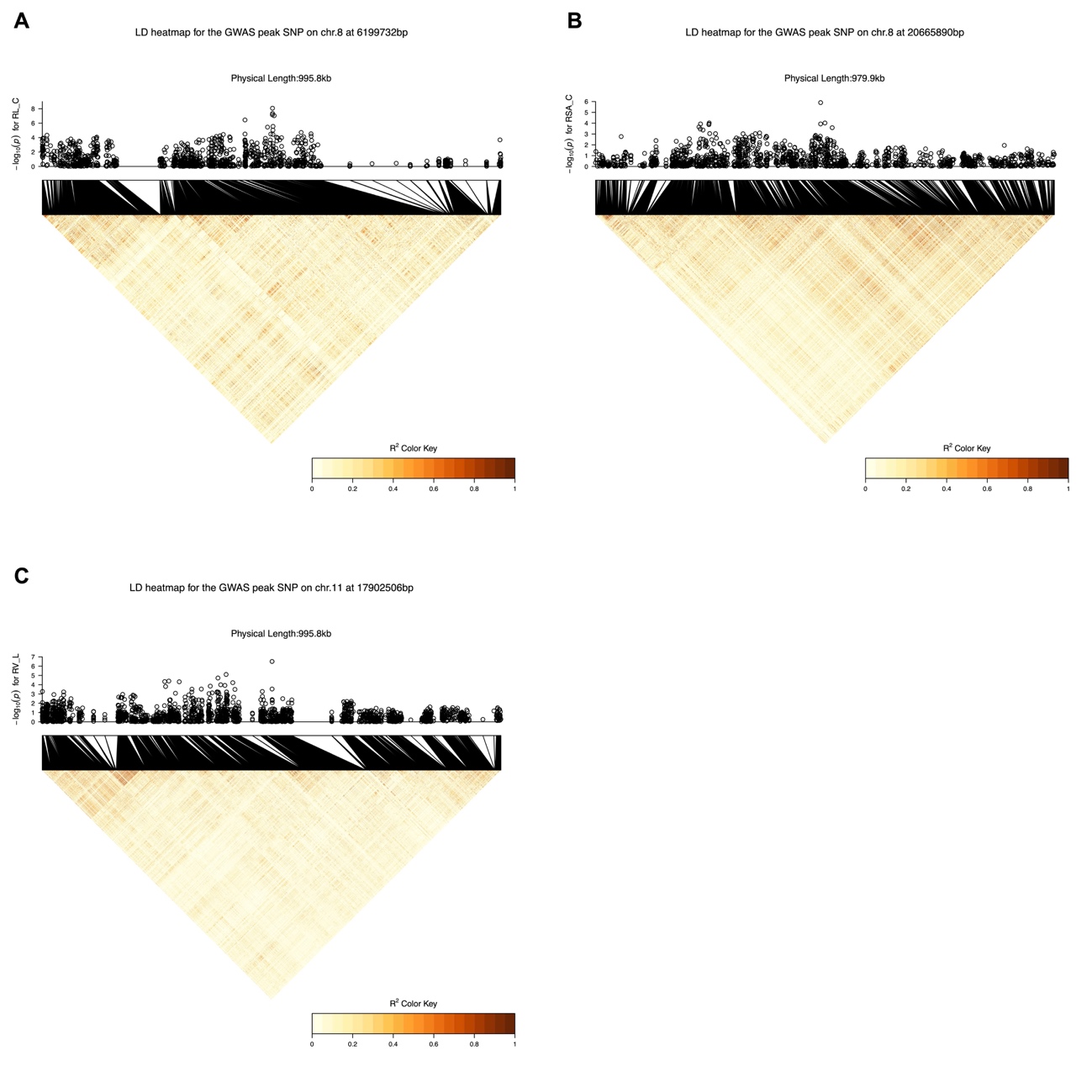


**Figure S5. LD heatmaps around the GWAS peak SNPs.** (A) LD heatmap for the GWAS peak SNP at 6,199,732 bp on chromosome 8 with the regional GWAS Manhattan plot for RL_C, (B) LD heatmap for the GWAS peak SNP at 20,665,890 bp on chromosome 8 with the regional GWAS Manhattan plot for RSA_C, and (C) LD heatmap for the GWAS peak SNP at 17,902,506 bp on chromosome 11 with the regional Manhattan plot for RV_L. In each plot, upper and lower panel show GWAS Manhattan plot and LD heatmap around peak SNP, respectively. The pairwise LD, shown as *R*^2^ in the figures, was calculated for all pairs of SNPs within 500 kb from the GWAS peak SNP and visualized using the {LDheatmap} package. The root phenotype with the lowest *P*-value for the GWAS peak SNP was selected as the representative result and shown in the Manhattan plot.

LD; linkage disequilibrium, GWAS; genome-wide association study, SNP; single nucleotide polymorphism, RL_C; crown root length, RSA_C; crown root surface area, RV_L; lateral root volume


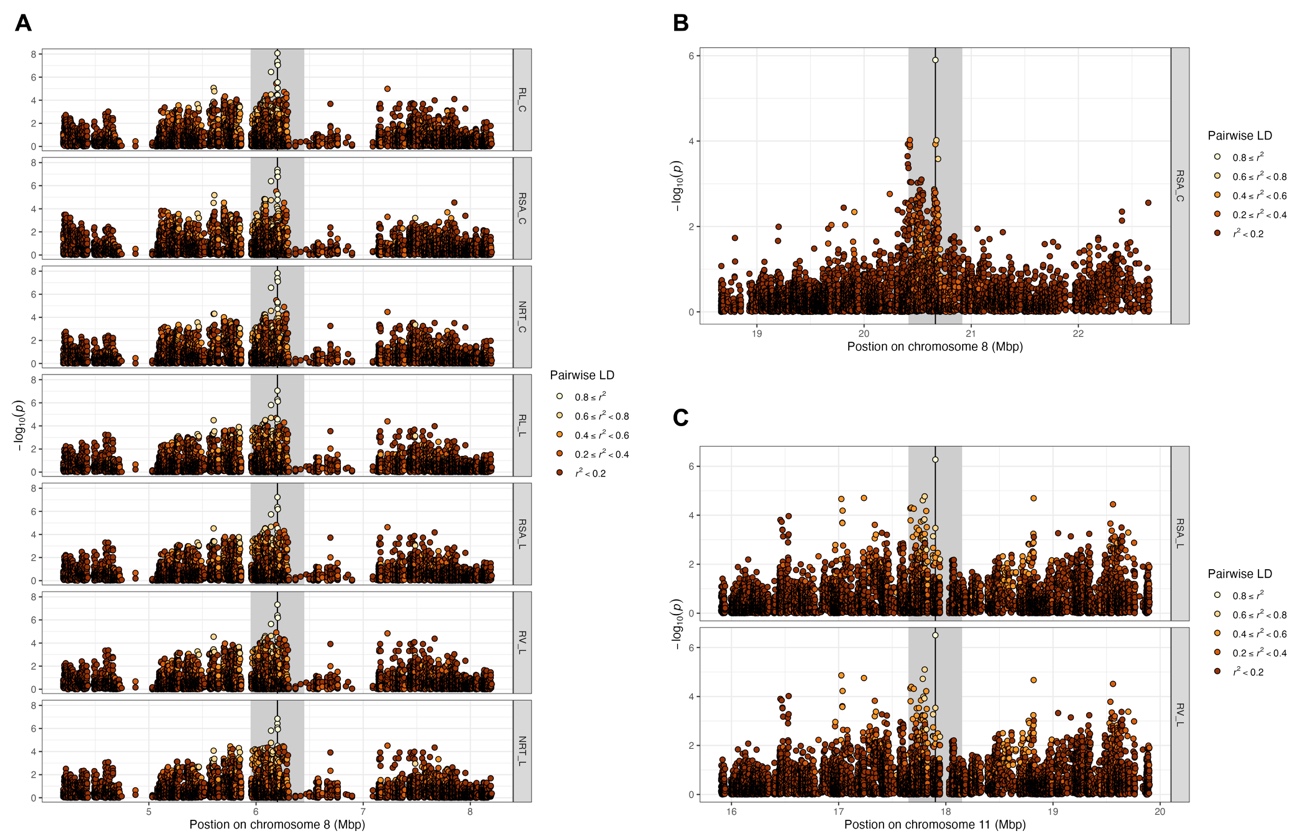


**Figure S6. Regional Manhattan plots colored by pairwise LD (*r*^2^) with the peak SNP.** (A) regional Manhattan plot for the GWAS peak SNP at 6,199,732 bp on chromosome 8, (B) regional Manhattan plot for the GWAS peak SNP at 20,665,890 bp on chromosome 8, and (C) regional Manhattan plot for the GWAS peak SNP at 17,902,506 bp on chromosome 11. The pairwise LD (*r*^2^ statistic) with the GWAS peak SNP was calculated for all SNPs within 2 Mb from the peak SNP. The gray shaded area represents the ± 250 kb region from the GWAS peak SNP.

LD; linkage disequilibrium, SNP; single nucleotide polymorphism, GWAS; genome-wide association study, RL_C; crown root length, RSA_C; crown root surface area, NRT_C; the number of crown root tips, RL_L; lateral root length, RSA_L; lateral root surface area, RV_L; lateral root volume, NRT_L; the number of lateral root tips


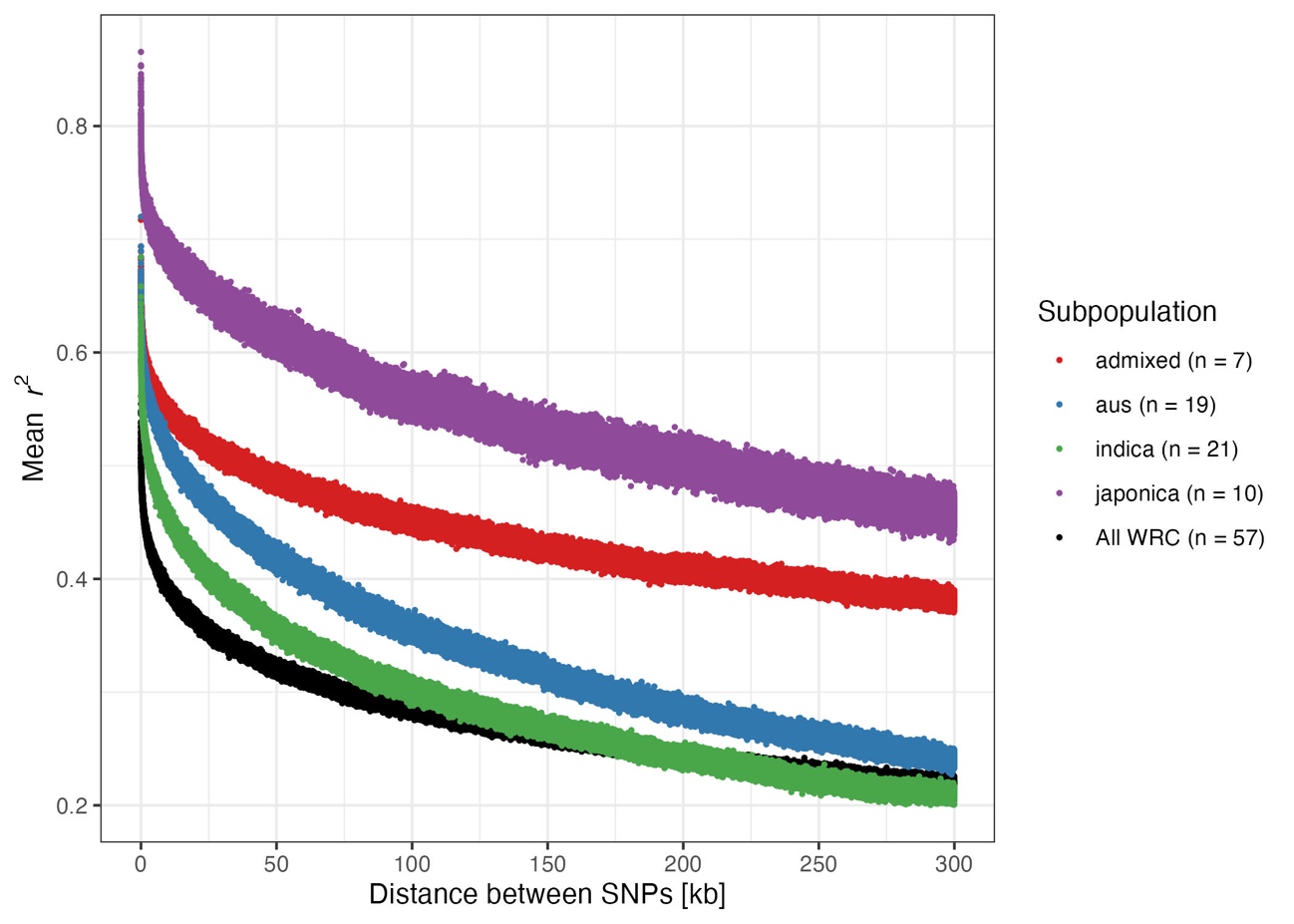


**Figure S7. Genome-wide LD decay.** The LD decay was calculated using the default method of the PopLDdecay software for all WRC lines and for each subpopulation.

LD; linkage disequilibrium, WRC; world rice core-collection


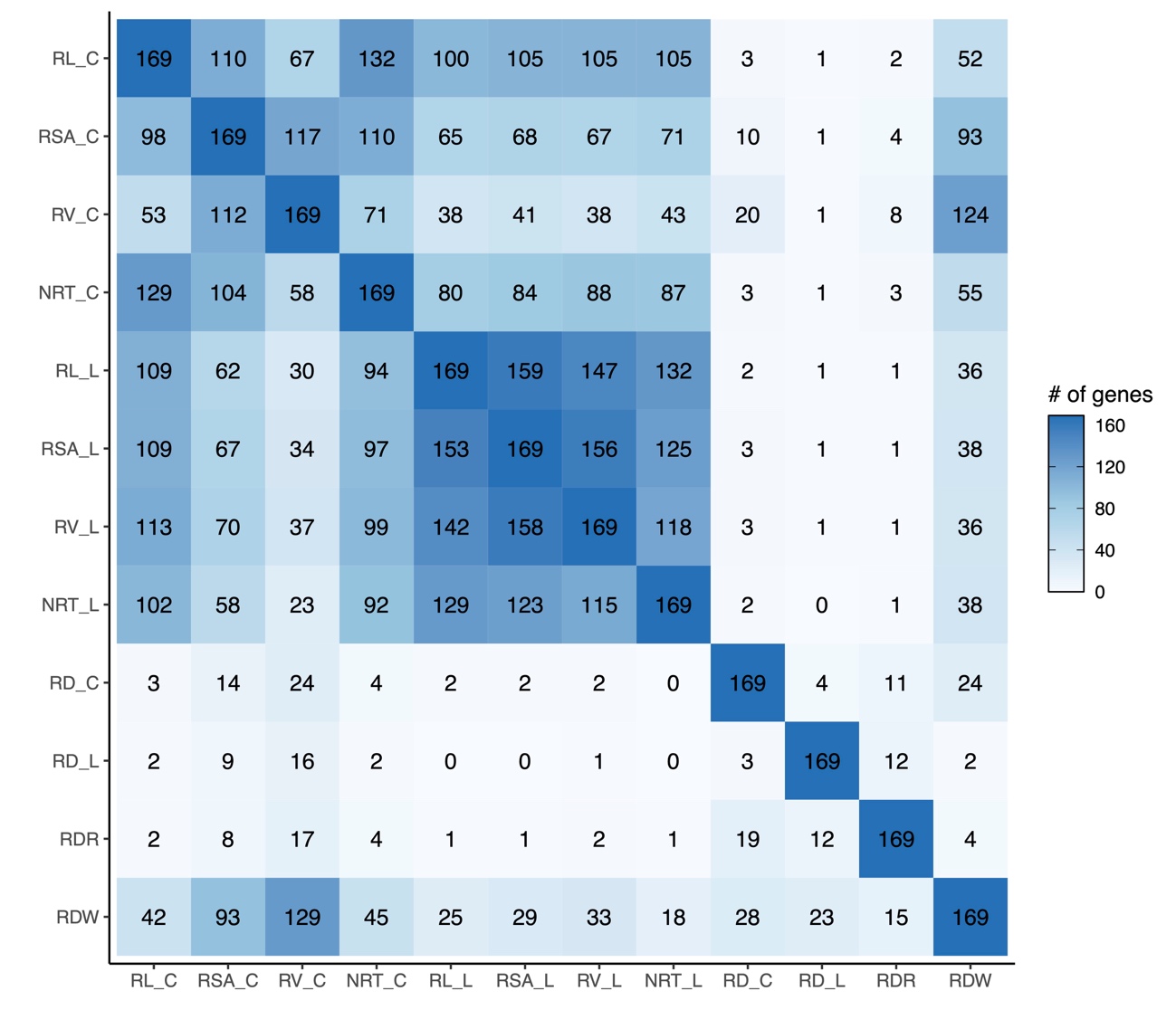
**Figure S8. The number of overlapped genes between the top 1% associations for different root phenotypes.** The numbers in the upper-right corner represent the number of overlapped genes in the top 1% positive associations, while the numbers in the lower-left corner represent the number of overlapped genes in the top 1% negative associations. For example, the largest overlap was observed between the top 1% positive associations for RL_L and RSA_L (159/169 genes were overlapped).

TWAS; transcriptome-wide association study, RL_C; crown root length, RSA_C; crown root surface area, RV_C; crown root volume, NRT_C; the number of crown root tips, RL_L; lateral root length, RSA_L; lateral root surface areas, RV_L; lateral root volume, NRT_L; the number of lateral root tips, RD_C; crown root diameter, RD_L; lateral root diameter, RDR; ratio of deep rooting, RDW; root dry weight

**
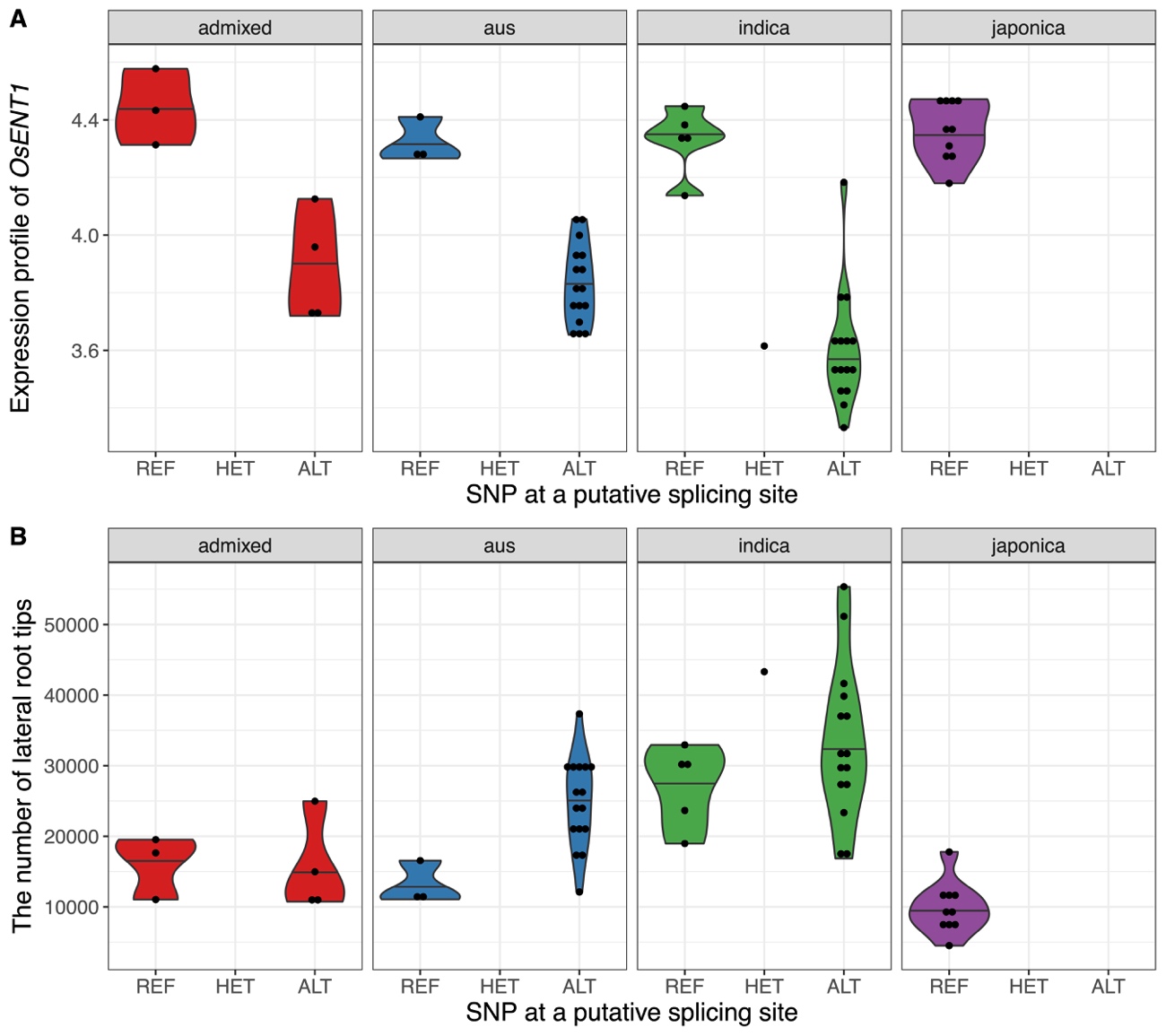
Figure S9. The dependence of (A) the expression profile of *OsENT1* and (B) the number of lateral root tips on the SNP at a putative splicing site of *OsENT1.*** The SNP in the x-axis was located at 6,143,760 bp on chromosome 8, which was 7 bp downstream of the first exon of *OsENT1* in the MU7 gene model. The horizontal line in the violin plot represents the median of the values.

SNP; single nucleotide polymorphism, LD; linkage disequilibrium, REF; reference allele homozygous, HET; heterozygous, ALT; alternative allele homozygous

**
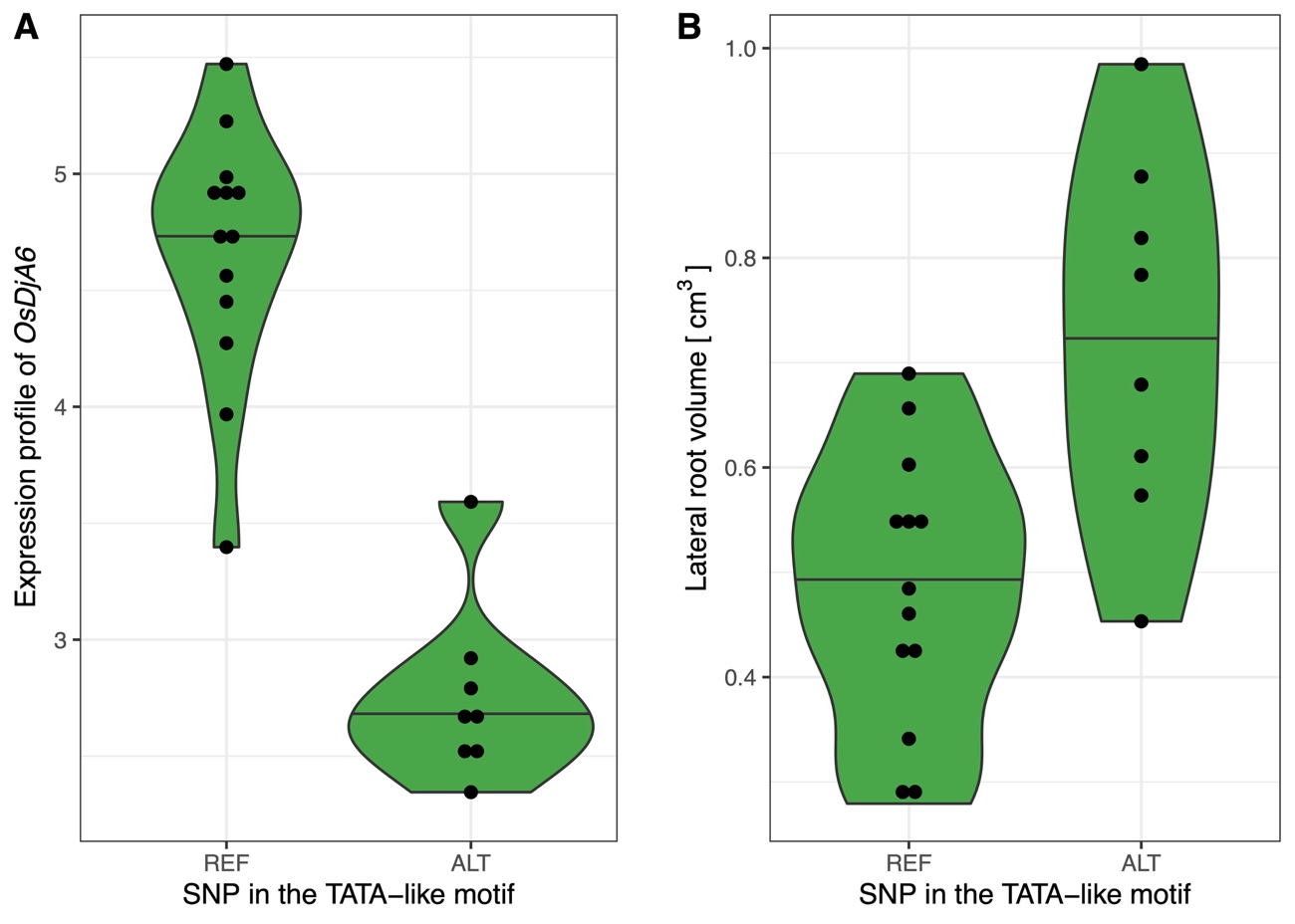
Figure S10. The dependence of (A) the expression profile of *OsDjA6* and (B) the lateral root volume on the SNP in a TATA box-like motif at an upstream of *OsDjA6* in *indica* subpopulation*.*** The SNP variant in the x-axis was located at 27,504,969 bp on chromosome 4, which was in a TATA box-like motif at an upstream of *OsDjA6* in the MU7 gene model. The horizontal line in the violin plot represents the median of the values.

SNP; single nucleotide polymorphism, REF; reference allele homozygous, ALT; alternative allele homozygous
